# Compatibility of insecticides and a phagostimulant with *Ganaspis kimorum*, a parasitoid of *Drosophila suzukii*

**DOI:** 10.3389/fpls.2026.1840796

**Published:** 2026-05-29

**Authors:** Jack Collins, Yahel Ben-Zvi, John McCulloch, Ashfaq A. Sial, Robert Holdcraft, Philip Fanning, Rufus Isaacs, Elena Rhodes, Oscar Liburd, Cesar Rodriguez-Saona

**Affiliations:** 1 Philip E. Marucci Center for Blueberry & Cranberry Research and Extension (PE) Marucci Center, Rutgers University, Chatsworth, NJ, United States; 2 Department of Entomology, University of Georgia, Athens, GA, United States; 3 School of Biology and Ecology, University of Maine, Orono, ME, United States; 4 Department of Entomology, Michigan State University, East Lansing, MI, United States; 5 Entomology and Nematology Department, University of Florida, Gainesville, FL, United States

**Keywords:** behavioral manipulation, biological control, Combi-protec^®^, integrated pest management, invasive pest, spotted-wing drosophila

## Abstract

**Introduction:**

Spotted-wing drosophila, *Drosophila suzukii* (Matsumura), is a globally invasive and economically important pest of thin-skinned fruits. Behavioral manipulation strategies, such as phagostimulants, are being explored to increase efficacy and reduce reliance on chemical control; however, these approaches may adversely affect non-target beneficial organisms.

**Methods:**

In laboratory and semi-field studies, we evaluated the lethal non-target effects of four conventional insecticides: phosmet (Imidan^®^), zeta-cypermethrin (Mustang^®^ Maxx), spinetoram (Delegate^®^), and cyantraniliprole (Exirel^®^); and two organic insecticides: pyrethrins (PyGanic^®^) and spinosad (Entrust^®^). These insecticides were applied alone or in combination with a commercial phagostimulant, Combi-protec^®^, and exposed to adult male and female *Ganaspis kimorum* Buffington, a larval parasitoid of *D. suzukii*. Insecticides were tested at three rates using two application methods: cover sprays, which treated the entire surface area, and bait sprays, which delivered discrete droplets to minimize overall exposure.

**Results:**

Among the insecticides evaluated, zeta-cypermethrin caused the highest mortality, whereas cyantraniliprole and pyrethrins caused the lowest; males were generally more sensitive than females. The addition of Combi-protec^®^ increased the lethality of ingestion-active insecticides (phosmet, spinetoram, and spinosad), but had little effect on zetacypermethrin, cyantraniliprole, and pyrethrins, suggesting that Combi-protec^®^ enhances feeding by *G. kimorum*. As expected, lower insecticide rates generally reduced mortality. Mortality was often lower when phosmet, spinetoram, and spinosad combined with Combi-protec^®^ were applied as bait sprays rather than cover sprays.

**Discussion:**

These findings indicate that careful insecticide selection and targeted application methods, in combination with phagostimulants and synchronized with adult *G. kimorum* activity, may reduce negative impacts on biological control and enhance integrated pest management programs.

## 1 Introduction

The spotted-wing drosophila, *Drosophila suzukii* (Matsumura) (Diptera: Drosophilidae), is an invasive pest native to East Asia that has become a major global threat to ripe and ripening thin-skinned and stone fruits, including blueberries, raspberries, strawberries, and cherries (Lee et al., 2011; Asplen et al., 2015; Rodriguez-Saona et al., 2019; Kirschbaum et al., 2020; Tait et al., 2021). Following its rapid spread across Europe, Africa, and the Americas in the 2010s, *D. suzukii* has caused substantial economic losses in fresh fruit production systems worldwide (DiGiacomo et al., 2019; Rodriguez-Saona et al., 2019; Yeh et al., 2020). Unlike most drosophilids, *D. suzukii* females possess a serrated ovipositor that enables them to pierce intact fruit skin and oviposit directly into the pulp. A single fruit may harbor dozens of eggs, and larval feeding results in extensive internal damage, rendering fruit unmarketable (Kirschbaum et al., 2020; Tait et al., 2021). As a result, *D. suzukii* poses a significant challenge to sustainable fruit production, necessitating the development of effective management strategies to minimize yield losses.

Currently, frequent applications of broad-spectrum insecticides remain the primary tool for suppressing *D. suzukii* populations (Diepenbrock et al., 2016; Tait et al., 2021). Modern insecticides provide many benefits to growers because they are fast-acting, highly effective, and readily deployable using existing application technologies (Sparks, 2013; Sparks et al., 2019). However, heavy reliance on these insecticides for *D. suzukii* management can accelerate the evolution of resistance, impact non-target organisms and the environment, and lead to secondary pest outbreaks (Gress and Zalom, 2019; Disi and Sial, 2021; Deans and Hutchison, 2022a, b). For example, resistance to organic spinosad has been reported in commercial raspberry plantings in California, USA (Gress and Zalom, 2019). Incorporating alternative strategies into *D. suzukii* management programs can provide additional options and promote more sustainable, holistic integrated pest management (IPM) approaches (Fitt, 2000; Dara, 2019; Theenoor et al., 2024).

Behavioral control strategies for *D. suzukii*, including the use of baits in combination with insecticides (Knight et al., 2016; Noble et al., 2022; Ferguson et al., 2025), offer opportunities to reduce reliance on conventional insecticides (Cloonan et al., 2018). Bait-insecticide formulations typically contain lower concentrations of active ingredients but maintain high efficacy by enhancing pest attraction and feeding (Knight et al., 2016; Babu et al., 2022). One such commercially available bait for *D. suzukii*, Combi-protec^®^ (Andermatt UK, Hove, UK), consists of sugars and plant extracts that function as phagostimulants, increasing feeding on treated surfaces (Babu et al., 2022; Ferguson et al., 2025). When used as an adjuvant, Combi-protec^®^ can significantly enhance insecticide efficacy at short ranges by exploiting *D. suzukii* feeding behavior (Babu et al., 2022; Noble et al., 2022). Research has demonstrated that combining Combi-protec^®^ with spinosad (Entrust^®^ 2SC Naturalyte) results in higher adult mortality and reduced progeny emergence compared with insecticide applications alone (Babu et al., 2022; Chandasir et al., 2022). Under field conditions, this approach can reduce the total amount of active ingredient applied per spray, lowering production costs and potentially slowing resistance development (Noble et al., 2021, 2022; Ferguson et al., 2025). In addition, bait-insecticide applications can be restricted to non-edible plant parts, such as stems and leaves, thereby reducing pesticide exposure risks for consumers and farm workers (Kalyabina et al., 2021). Therefore, behavioral manipulation should be explored as a potential tool for inclusion in integrated pest management programs for managing *D. suzukii*.

Classical biological control represents another promising alternative strategy. In invaded regions, resident natural enemies generally fail to exert sufficient top-down pressure on *D. suzukii*, largely due to their inability to overcome the fly’s immune defenses (Kacsoh and Schlenke, 2012; Poyet et al., 2013). This limitation has motivated the exploration of parasitoids that coevolved with *D. suzukii* in its native range. One such parasitoid is *Ganaspis kimorum* Buffington (Hymenoptera: Figitidae) (formerly known as *G. brasiliensis* Ihering “G1” strain), which has been recently approved for release in North America due to its narrow host range (Daane et al., 2016; Giorgini et al., 2019; Seehausen et al., 2020). Since 2022, mass rearing and releases of *G. kimorum* have been initiated across the United States to establish this species (Stahl et al., 2024). In its native range, *G. kimorum* achieves high parasitism rates and has the potential to exert similar top-down pressure in invaded regions (Girod et al., 2018; Wang et al., 2020; Ben-Zvi and Rodriguez-Saona, 2025), providing a complement to chemical and behavioral control.

Despite their promise, integrating biological and behavioral control strategies with chemical control may result in antagonistic interactions that limit overall effectiveness. Commonly used insecticides can negatively affect non-target organisms, including *G. kimorum* (Sarkar et al., 2019; Fellin et al., 2024; Lisi et al., 2024a). Exposure to these compounds may cause mortality in released parasitoids (Roubos et al., 2019), hindering their establishment and persistence. Furthermore, adult parasitoids consume sugars (Lee, 2024) and may respond to phagostimulants such as Combi-protec^®^ in ways similar to *D. suzukii*. Consequently, although both behavioral and biological control approaches have potential benefits, their interactions with chemical control must be carefully evaluated to avoid unintended non-target effects.

Here, we hypothesized that insecticide compatibility with *G. kimorum* depends on mode of action, application method, and interaction with phagostimulants. Laboratory and semi-field bioassays were conducted to quantify *G. kimorum* mortality following exposure to conventional and organic insecticides, with or without the addition of Combi-protec^®^. Specifically, we asked: (1) Do insecticides differ in their lethality to *G. kimorum*, and are these effects influenced by sex, insecticide rate, and residual activity? (2) Does the phagostimulant Combi-protec^®^ increase the lethality of insecticides to *G. kimorum*? (3) Is insecticide lethality, with and without Combi-protec^®^, influenced by application method (cover versus bait spray)? (4) Do laboratory results translate to field conditions? Collectively, these findings provide guidance for insecticide selection when integrating novel behavioral control strategies, helping to minimize unintended mortality of biological control agents and reduce barriers to their establishment.

## 2 Materials and methods

### 2.1 Insects

Wasps were reared following the protocol described by Rossi-Stacconi et al. (2022). To maintain the colony, store-bought blueberries were rinsed with water and dried then exposed to *D. suzukii* for 48–72 h in 0.4 × 0.4 × 0.5 m plexiglass cages, after which the infested fruits were provided to *G. kimorum* adults for 96–144 h. The berries were then kept in 473 mL plastic deli cups with screened lids at 22 ± 1 °C, 55% RH, and 16:8 L:D photoperiod until wasp emergence. Before being used in bioassays, wasps were maintained in groups of 250 individuals (approx. 1:1 male: female) in 50 mL polystyrene vials (Genesee Scientific, San Diego, CA, USA) and held in an incubator (Percival Scientific, Perry, IA, USA) under controlled conditions (23 ± 2 °C, 60–70% RH, and 16:8 h L:D photoperiod). All wasps used in the bioassays were 5–15 days old, which falls within the lower range of their average adult lifespan (Wang et al., 2018). This age range has been used in previous experiments and is not expected to significantly affect the results; it also corresponds to the peak reproductive window with minimal physiological variation (Ben-Zvi and Rodriguez-Saona, 2025). Prior to each bioassay, wasps were starved for approximately 12 h.

### 2.2 Insecticides

Six insecticides—four conventional and two organic—were evaluated in the bioassays. The active ingredients represented four insecticide classes with distinct modes of action (Sparks and Nauen, 2015): organophosphates (IRAC Group 1B), pyrethroids/pyrethrins (IRAC Group 3A), spinosyns (IRAC Group 5), and anthranilic diamides (IRAC Group 28). Although these insecticide classes may exhibit both contact and ingestion activity, organophosphates and pyrethroids/pyrethrins act predominantly through contact, whereas spinosyns and diamides are primarily effective through ingestion. The insecticides tested included the organophosphate phosmet (Imidan^®^ 70-W; Gowan Company, Yuma, AZ, USA); the pyrethroid zeta-cypermethrin (Mustang^®^ Maxx; FMC Corporation, Philadelphia, PA, USA); the pyrethrin PyGanic^®^ EC (MGK, Minneapolis, MN, USA); the conventional spinosyn spinetoram (Delegate^®^ WG; Corteva, Johnston, IA, USA); the organic spinosyn spinosad (Entrust^®^ SC; Corteva); and the diamide cyantraniliprole (Exirel^®^; FMC Corporation) (**Table 1**). All insecticides are commercially available and were selected based on their relevance to *D. suzukii* management in conventional and organic small fruit production systems (Shawer et al., 2018; Tait et al., 2021). According to Tait et al. (2021), these insecticides are ranked by lethality to *D. suzukii* (highest to lowest) as follows: zeta-cypermethrin = phosmet > cyantraniliprole = spinetoram > spinosad > pyrethrins. All are rated good to excellent, except pyrethrins, which are considered weak to fair. Pyrethrins and spinosad are approved for organic production by the Organic Materials Review Institute (OMRI). Grandevo^®^ (*Chromobacterium subtsugae* strain PRAA4-1^T^) was also tested, but it showed minimal to no toxicity toward *G. kimorum* and is of only minor importance in *D. suzukii* management on organic farms. Therefore, those results were not included in this paper.

**Table 1:** Active ingredient (a.i.), insecticide brand name, formulation, percent of a.i. per insecticide, insecticide class, type, and insecticide resistance action committee (IRAC), and full, half, and quarter rates of cover spray.

Active ingredient	Brand name	Formulation	a.i. %	Class	Type	IRAC	Full rate a.i. mg/100 mL distilled water ^a^	Half rate a.i. mg/100 mL distilled water	Quarter rate a.i. mg/100 mL distilled water
Phosmet	Imidan^®^ 70-W	Wettable powder	70%	Organophosphate	Conventional	1B	222.60	111.30	55.65
Zeta-cypermethrin	Mustang^®^ Maxx	Emulsifiable concentrate	9.15%	Pyrethroid	Conventional	3A	7.54	3.77	1.88
Pyrethrins	PyGanic^®^ EC	Emulsifiable concentrate	1.4%	Pyrethrin	Organic	3A	2.87	1.43	0.72
Spinetoram	Delegate^®^ WG	Water dispersible granule	25%	Spinosyn	Conventional	5	22.50	11.25	5.63
Spinosad	Entrust^®^ SC	Suspension concentrate	22.5%	Spinosyn	Organic	5	10.83	5.41	2.71
Cyantraniliprole	Exirel^®^	Suspoemulsion	10.2%	Anthranilic diamide	Conventional	28	34.53	17.26	8.63

^a^The amount of active ingredient (a.i.) was calculated by multiplying the concentration of each insecticide per 100 mL of distilled water by the percentage of a.i. indicated on the product label.

### 2.3 Treatment formulations

Two application methods were used to apply insecticide treatments: a cover spray and a bait spray. Cover sprays consisted of 2 mL of formulation applied with a Potter precision spray tower (Burkard Scientific, United Kingdom), delivering a spray volume equivalent to 468 L/ha (50 gal/acre). Bait sprays consisted of a uniform array of twenty 10 µL droplets dispensed with a pipette, following the procedures described by Mermer et al. (2019) and Noble et al. (2022).

Formulations were prepared using the maximum labeled field rates of 1.49 kg/ha (1.33 lb/acre) for phosmet, 0.292 L/ha (4.0 fl oz/acre) for zeta-cypermethrin, 1.14 L/ha (15.6 fl oz/acre) for pyrethrins, 0.42 kg/ha (6.0 oz/acre) for spinetoram, 0.438 L/ha (6.0 fl oz/acre) for spinosad, and 0.987 L/ha (13.5 fl oz/acre) for cyantraniliprole. For Combi-protec^®^, we used the label rate of 1.12 kg/ha (1 lb/acre). Based on these rates, the full (high)-rate concentrations per 100 mL of distilled water were: 318 mg for phosmet, 0.062 mL for zeta-cypermethrin, 0.244 mL for pyrethrins, 90 mg for spinetoram, 0.094 mL for spinosad, and 0.210 mL for cyantraniliprole. These concentrations were calculated by dividing the maximum labeled field rates by the spray volume (468 L/ha) used in the spray tower and scaling to 100 mL. For example, the full rate of phosmet was calculated by dividing 1.49 kg/ha by 468 L/ha and multiplying by 100 mL. To facilitate comparison, we converted the liquid zeta-cypermethrin, pyrethrins, spinosad, and cyantraniliprole into mg using their reported densities (1.329 g/cm³, 0.84 g/cm³, 0.512 g/cm³, 1.612 g/cm³, respectively). These values were then used to calculate the amount of active ingredient (a.i.) for each insecticide used in the laboratory bioassays (**Table 1**). Half-rate (intermediate) and quarter-rate (low) formulations were prepared by diluting the full-rate solutions in proportion. To maintain a consistent concentration of Combi-protec^®^ across treatments, 0.24 g was added per 100 mL of solution at each insecticide rate. For bait sprays, three application rates were used: 20 droplets (100%, high), 10 droplets (50%, intermediate), and 2 droplets (10%, low). Each droplet had an equal concentration, and the rates were based on the number of droplets.

Each laboratory bioassay (see below) run included a control treatment. For insecticide-only treatments, the control consisted of distilled water applied either as a 2 mL cover spray using the Potter spray tower or as twenty 10 µL water droplets for bait sprays. For treatments containing Combi-protec^®^, the control consisted of 0.24 g of Combi-protec^®^ dissolved in 100 mL of distilled water, applied using the same methods.

In addition to application method and rate, treatments were evaluated across three residue ages: 0, 3, and 7 days after treatment (DAT). These residue ages were selected to assess insecticide residual activity, reflecting the typical 7-day reapplication interval used by growers.

### 2.4 Laboratory bioassays

Bioassays were conducted to assess *G. kimorum* mortality after insecticide treatments with and without Combi-protec^®^, using methods described by Roubos et al. (2019). Wasps were briefly anesthetized with carbon dioxide prior to placement in experimental arenas. Each arena consisted of a 100 mm disposable plastic Petri dish (VWR, Radnor, PA, USA) containing five male or five female wasps, a cotton ball soaked in a 10% honey-water solution to reduce mortality in the control, and the treatment formulation (see below) applied to the underside of the dish lid. Treated lids were allowed to dry for 2 h before wasps were introduced. Each treatment was replicated 10 times.

Mortality was assessed 24 h post-exposure, and wasps were classified as alive, moribund (i.e., individuals that responded to probing but lacked vitality and vigor), or dead. Because few wasps were categorized as moribund and they were considered functionally dead, moribund and dead individuals were pooled prior to analysis. Adjusted percent mortality was calculated using Abbott’s formula (Abbott, 1925):


$$Adjusted\;Mortality\;(\%)=\;(\frac{M_{t}-M_{c}}{100-M_{c}})\times 100$$

Where *M_t_* is the percent mortality in the treatment group and *M_c_* is the percent mortality in the control group.

### 2.5 Semi-field bioassays

In addition to laboratory bioassays, semi-field bioassays were conducted in New Jersey and Georgia (USA) using three insecticides representing different modes of action and toxicity levels against *G. kimorum* (see Results). The insecticides were applied at their maximum labeled rates: zeta-cypermethrin (0.292 L/ha), spinosad (0.438 L/ha), and cyantraniliprole (0.987 L/ha). These semi-field bioassays provide a more realistic yet controlled assessment, using unsprayed fields, standardized treatment methods, and controlled insect exposure to insecticides, bridging the gap between laboratory and field studies. Treatments included each insecticide applied with and without Combi-protec^®^ (1.12 kg/ha), Combi-protec^®^ alone, and an untreated control (water only). All products were applied to individual blueberry bushes (N = 5) at their maximum labeled rates, with each bush receiving 125.1 mL of spray solution using a CO_2_ backpack sprayer (R&D Sprayer; Opelousas, LA) calibrated to 30 psi. The experiment followed a randomized complete block design with five replicates. Within blocks, treated bushes were separated by two untreated bushes, and blocks were separated by one row of bushes.

Applications were made early in the morning to avoid windy conditions and on sunny days (25–30 °C, 70–80% RH) during fruit ripening (July 2025). No to little rainfall was recorded during the experiment. Rainfall occurred after the 3 DAT sampling in sites sprayed with spinosad and zeta-cyantraniliprole. Bushes were sampled at 0, 3, and 7 DAT. A terminal sprig containing four to five leaves was collected from each bush, placed in a florist’s water pick, and transferred to a 946 mL (32 oz) ventilated clear plastic container. Fifteen blueberries from the same bush were added to each container. Samples were transported to the laboratory, where a cotton-plugged vial of water provided a water source for *G. kimorum*. Ten adult wasps (five males and five females, each between 5–15 days old) were introduced into each container. Containers were held in an environmental chamber at approximately 25 °C, 60% RH, and a 16:8 (L:D) photoperiod. Male and female wasps were assessed for mortality and moribundity at 24 and 72 h after exposure, with dead and moribund individuals combined for analysis. Adjusted percent mortality was calculated using Abbott’s formula as described above.

### 2.6 Statistical analysis

To address our first question—whether insecticides differ in lethality to *G. kimorum* and whether this varies by sex, insecticide rate, or DAT—we conducted generalized linear models (GLMs) in RStudio (v4.2; R Core Team, 2024) after confirming non-normality with Shapiro-Wilk tests. All GLMs assumed gamma distributions with a log link, and data were shifted by 1 prior to analysis. Following the GLMs, we performed *post hoc* tests of estimated marginal means. The response variable was Abbott-adjusted mortality, and the explanatory variables were insecticide type, insecticide rate, DAT, and their interactions. Separate models were run for each combination of *G. kimorum* sex and application method (cover spray or bait spray). For this first question, only treatments without Combi-protec^®^ were included in the analysis.

Our second question examined how the inclusion of Combi-protec^®^ influenced insecticide lethality. In these GLMs, explanatory variables included insecticide type, inclusion or exclusion of Combi-protec^®^, insecticide rate, DAT, and their interactions. Separate models were again conducted for each combination of *G. kimorum* sex and application method.

Next, we evaluated the influence of application method (cover spray vs. bait spray) on insecticide lethality, both with and without Combi-protec^®^. In these models, explanatory variables were insecticide type, inclusion or exclusion of Combi-protec^®^, application method, insecticide rate, DAT, and their interactions. Separate models were conducted for each *G. kimorum* sex. Because application rates differed between methods (1×, ½×, and ¼× for cover spray; 100%, 50%, and 10% for bait spray), rates were grouped into high, intermediate, and low categories for comparison.

Finally, we analyzed field-sprayed material using GLMs with Abbott-adjusted mortality as the response variable, calculated separately for each insecticide trial. Explanatory variables included insecticide type, inclusion or exclusion of Combi-protec^®^, DAT, and their interactions. Separate models were conducted for each combination of *G. kimorum* sex and assessment interval (24 or 72 h after exposure).

## 3 Results

### 3.1 Do insecticides differ in their lethality to *G. kimorum*, and are these effects influenced by sex, insecticide rate, and residual activity?

**Figure 1:**
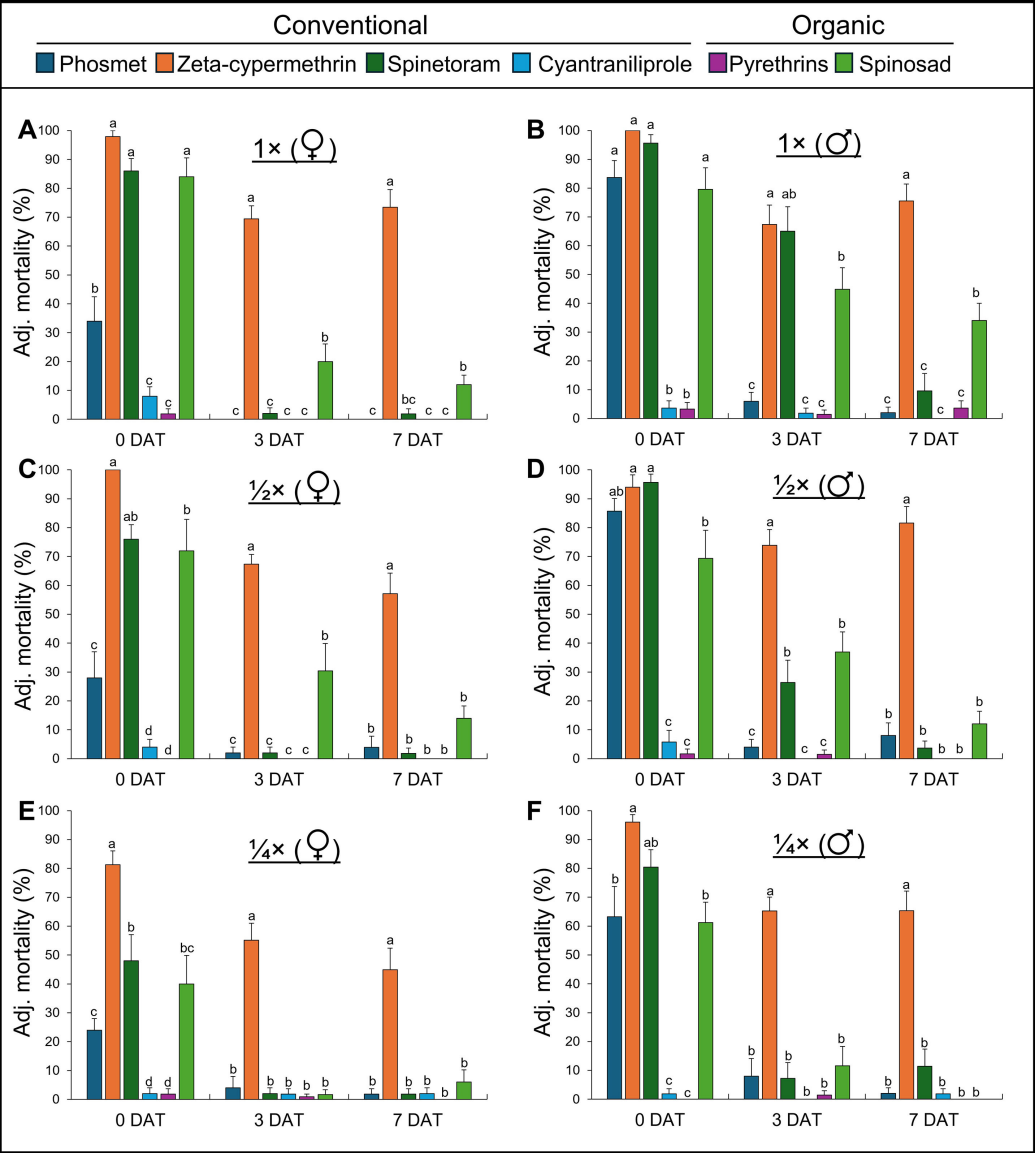
Effects of cover sprays at full **(A, B)**, half **(C, D)**, and quarter **(E, F)** rates and days after treatment (DAT) on female **(A, C, E)** and male **(B, D, F)** *Ganaspis kimorum* 24 h mortality. Letters indicate a significant difference between treatments (*p* < 0.05).

**Figure 2:**
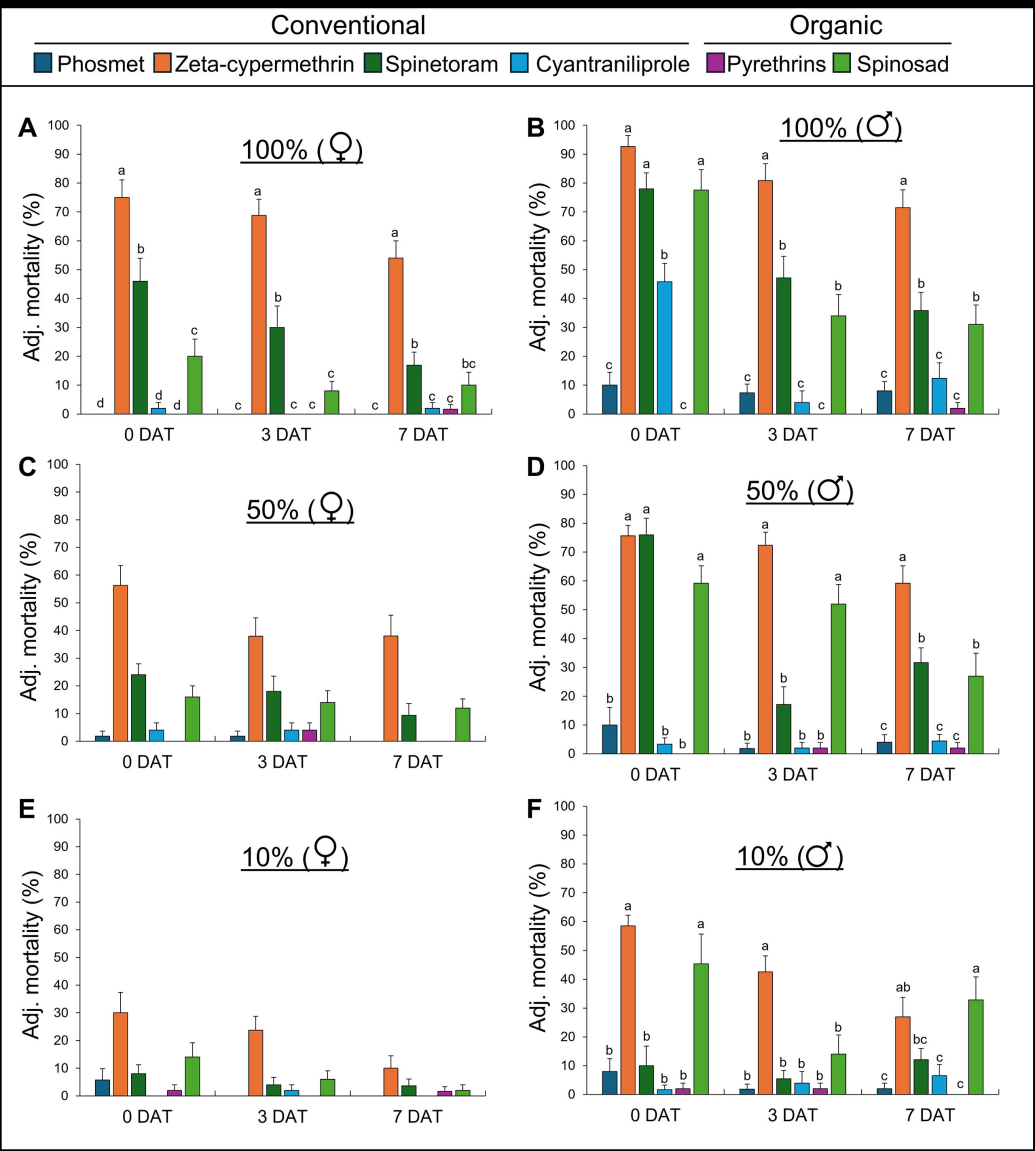
Effects of bait sprays at 100% **(A, B)**, 50% **(C, D)**, and 10% **(E, F)** and days after treatment (DAT) on female **(A, C, E)** and male **(B, D, F)** *Ganaspis kimorum* 24 h mortality. Letters indicate a significant difference between treatments (*p* < 0.05).

Lethality of insecticides on *G. kimorum* was generally greater at higher application rates and earlier DATs, and males were more susceptible to insecticides than females (**Figures 1**, **2**). For both application methods, zeta-cypermethrin consistently caused the highest mortality of *G. kimorum* across all rates, DATs, and both sexes, averaging approximately 80% mortality in cover sprays and 60% in bait sprays (**Tables 2**, **3**; **Figures 1**, **2**). Following zeta-cypermethrin, spinetoram and spinosad also caused high mortality, particularly at earlier DATs. Phosmet likewise resulted in high mortality, especially in males, when applied as a cover spray at 0 DAT (**Figure 1**). In contrast, cyantraniliprole and pyrethrins produced very low mortality, averaging less than 10%. The only exception for cyantraniliprole was in males exposed to the high-rate bait spray, which reached an average of 20% mortality across the three DATs (**Figure 2**).

**Table 2:** Effects of insecticide type, rate, and days after treatment (DAT) on mortality of *Ganaspis kimorum* after 24 h exposure to a cover spray (without Combi-protec^®^) in the laboratory.

Variables	χ^2^		df	*P* ^a^	
	Male	Female		Male	Female
Insecticide	352.03	247.71	5	**<0.001**	**<0.001**
Rate	3.77	27.03	2	0.152	**<0.001**
DAT	0.152	93.20	2	**<0.001**	**<0.001**
Insecticide × Rate	7.66	32.41	10	0.662	**<0.001**
Insecticide × DAT	156.19	74.77	10	**<0.001**	**<0.001**
Rate × DAT	52.26	18.24	4	**<0.001**	**<0.001**
Insecticide × Rate × DAT	70.37	29.29	20	**<0.001**	0.082

^a^Bold indicates significant P-value (*p* < 0.05).

**Table 3:** Effects of insecticide type, insecticide rate, and days after treatment (DAT) on mortality of *Ganaspis kimorum* after 24 h of exposure to a bait spray (without Combi-protec^®^) in the laboratory.

Variables	χ^2^		df	*P* ^a^	
	Male	Female		Male	Female
Insecticide	137.89	50.47	5	**<0.001**	**<0.001**
Rate	101.47	48.95	2	**<0.001**	**<0.001**
DAT	1.36	1.12	2	0.506	0.573
Insecticide × Rate	102.84	69.64	10	**<0.001**	**<0.001**
Insecticide × DAT	30.93	13.56	10	**<0.001**	0.194
Rate × DAT	34.85	9.16	4	**<0.001**	0.057
Insecticide × Rate × DAT	85.58	20.43	20	**<0.001**	0.431

^a^Bold indicates significant P-value (*p* < 0.05).

### 3.2 Does the phagostimulant Combi-protec^®^ increase the lethality of insecticides to *G. kimorum*?

**Figure 3:**
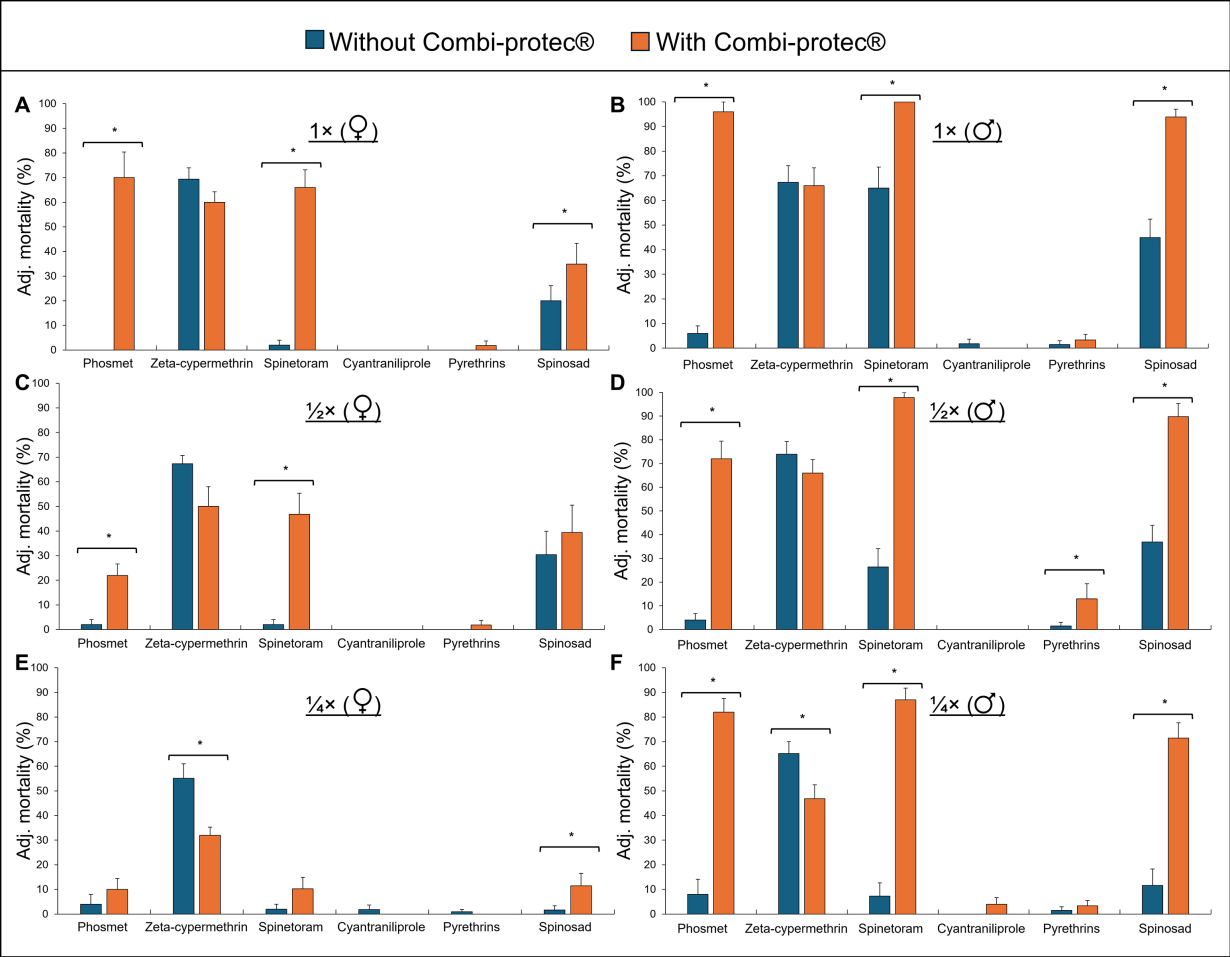
Effects of cover sprays at full **(A, B)**, half **(C, D)**, and quarter **(E, F)** rates and Combi-protec^®^ on female **(A, C, E)** and male **(B, D, F)** *Ganaspis kimorum* 24 h mortality at 3 days after treatment (DAT). Asterisks (*) indicate a significant difference between treatments (*p* < 0.05).

**Figure 4:**
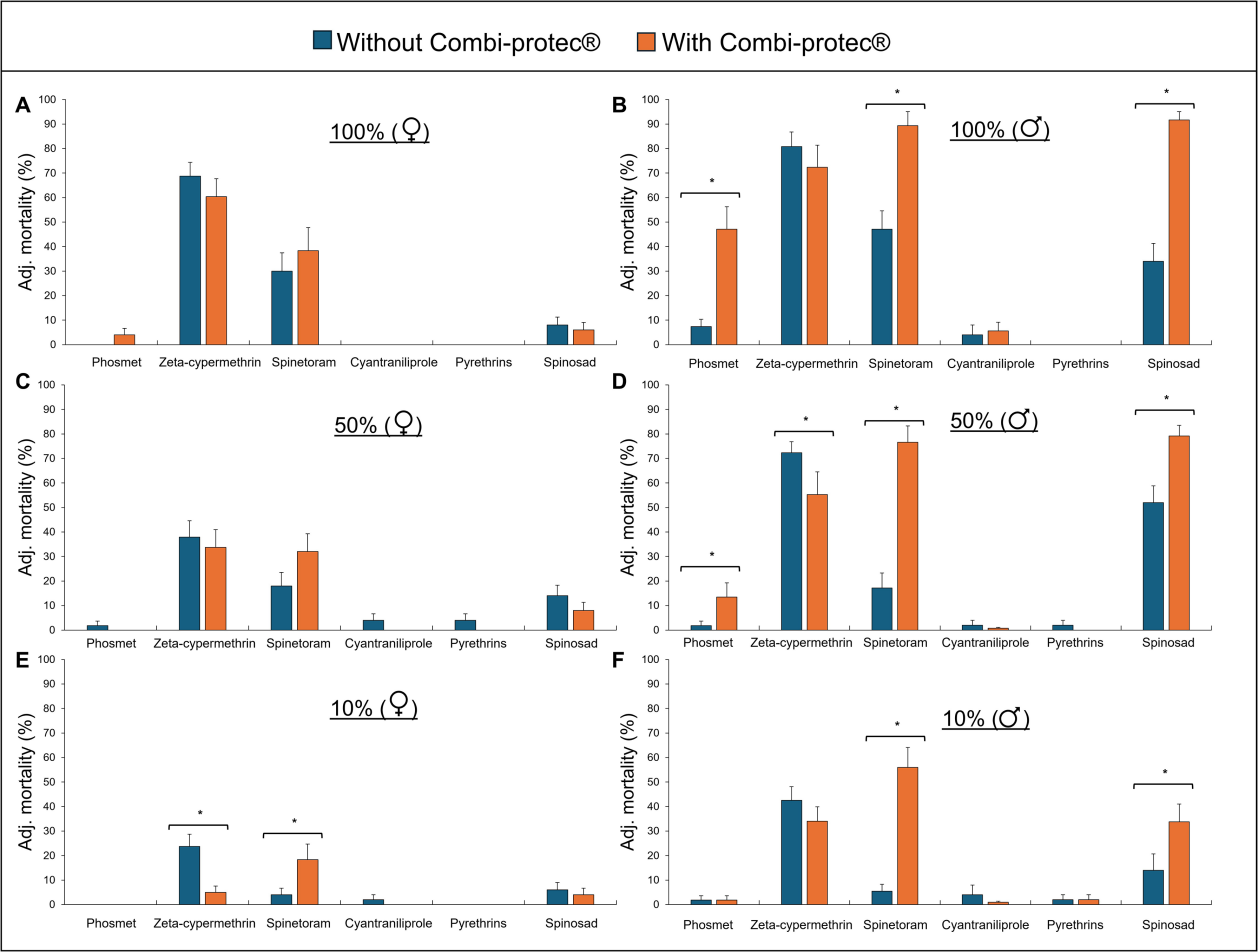
Effects of bait sprays at 100% **(A, B)**, 50% **(C, D)**, and 10% **(E, F)** and Combi-protec^®^ on female **(A, C, E)** and male **(B, D, F)** *Ganaspis kimorum* 24 h mortality at 3 days after treatment (DAT). Asterisks (*) indicate a significant difference between treatments (*p* < 0.05).

Lethality of insecticides on male and female *G. kimorum* applied as cover sprays, with and without Combi-protec^®^, at 0, 3, and 7 DAT is shown in **Supplementary Figures 1, 3**; **Figure 3**, respectively, whereas bait spray data are shown in **Supplementary Figure 2** (0 DAT), **Figure 4** (3 DAT), and **Supplementary Figure 4** (7 DAT). The addition of the phagostimulant Combi-protec^®^ significantly increased insecticide lethality in 52 of 216 instances (24%), calculated across 6 insecticides, 2 sexes, 3 rates, 2 spray methods, and 3 DATs (**Tables 4**, **5**; **Figures 3**, **4**; **Supplementary Figures 1**–**4**). However, the magnitude of this effect varied with insecticide rate, DAT, and the sex of *G. kimorum*. When applied as a cover spray, Combi-protec^®^ significantly enhanced the lethality of phosmet in 13 instances, spinetoram in 12 instances, and spinosad, particularly at later DATs, in 9 instances (**Figure 3**; **Supplementary Figures 1**, **3**). It also increased male mortality at low rates of pyrethrins applied as cover sprays (2 instances; **Supplementary Figure 3**). Similarly, under bait spray applications, Combi-protec^®^ significantly increased the lethality of phosmet in four instances, spinetoram in five instances, and spinosad in seven instances (**Figure 4**; **Supplementary Figures 2**, **4**).

**Table 4:** Effects of insecticide type, Combi-protec^®^, rate, and days after treatment (DAT) on mortality of *Ganaspis kimorum* after 24 h of exposure to a cover spray in the laboratory.

Variables	χ^2^		df	*P* ^a^	
	Male	Female		Male	Female
Insecticide	338.49	184.92	5	**<0.001**	**<0.001**
Combi-protec	4.44	28.43	1	**0.035**	**<0.001**
Rate	3.63	20.18	2	0.163	**<0.001**
DAT	147.37	69.57	2	**<0.001**	**<0.001**
Insecticide × Combi-protec	9.88	27.50	5	0.077	**<0.001**
Insecticide × Rate	7.37	24.19	10	0.690	**0.007**
Combi-protec × Rate	1.83	11.31	2	0.400	**0.004**
Insecticide × DAT	150.19	55.82	10	**<0.001**	**<0.001**
Combi-protec × DAT	55.09	7.74	2	**<0.001**	**0.021**
Rate × DAT	50.25	13.62	4	**<0.001**	**0.009**
Insecticide × Combi-protec × Rate	12.92	16.47	10	0.228	0.087
Insecticide × Combi-protec × DAT	129.53	22.25	10	**<0.001**	**0.014**
Insecticide × Rate × DAT	67.66	21.87	20	**<0.001**	0.348
Combi-protec × Rate × DAT	19.43	51.87	4	**<0.001**	**<0.001**
Insecticide × Combi-protec × Rate × DAT	35.53	46.21	20	**0.017**	**<0.001**

^a^Bold indicates significant P-value (*p* < 0.05).

**Table 5:** Effects of insecticide type, Combi-protec^®^, rate, and days after treatment (DAT) on mortality of *Ganaspis kimorum* after 24 h of exposure to a bait spray in the laboratory.

Variables	χ^2^		df	*P* ^a^	
	Male	Female		Male	Female
Insecticide	125.65	43.22	5	**<0.001**	**<0.001**
Combi-protec	22.46	0.19	1	**<0.001**	0.664
Rate	92.47	41.92	2	**<0.001**	**<0.001**
DAT	1.24	0.96	2	0.538	0.620
Insecticide × Combi-protec	35.71	5.23	5	**<0.001**	0.388
Insecticide × Rate	93.71	59.64	10	**<0.001**	**<0.001**
Combi-protec × Rate	21.82	2.28	2	**<0.001**	0.320
Insecticide × DAT	28.19	11.61	10	**0.002**	0.312
Combi-protec × DAT	20.47	2.94	2	**<0.001**	0.230
Rate × DAT	31.76	7.84	4	**<0.001**	0.098
Insecticide × Combi-protec × Rate	68.27	23.61	10	**0.005**	**0.009**
Insecticide × Combi-protec × DAT	25.23	19.67	10	**0.005**	**0.033**
Insecticide × Rate × DAT	77.99	17.50	20	**<0.001**	0.621
Combi-protec × Rate × DAT	19.50	2.59	4	**<0.001**	0.629
Insecticide × Combi-protec × Rate × DAT	63.48	24.92	20	**<0.001**	0.205

^a^Bold indicates significant P-value (*p* < 0.05).

In 18 instances (8%), however, Combi-protec^®^ significantly reduced insecticide efficacy. For example, it decreased the lethality of zeta-cypermethrin in 12 instances—particularly at later DATs—resulting in up to 50% lower *G. kimorum* mortality compared to zeta-cypermethrin applied alone (**Figures 3**, **4**; **Supplementary Figures 1**-**4**). At 0 DAT, Combi-protec^®^ also reduced spinosad lethality in four instances when applied as a cover spray (**Supplementary Figure 1**) and decreased cyantraniliprole lethality to males once at the high-rate bait spray (**Supplementary Figure 2**).

### 3.3 Is insecticide lethality, with and without Combi-protec^®^, influenced by application method (cover versus bait spray)?

**Figure 5:**
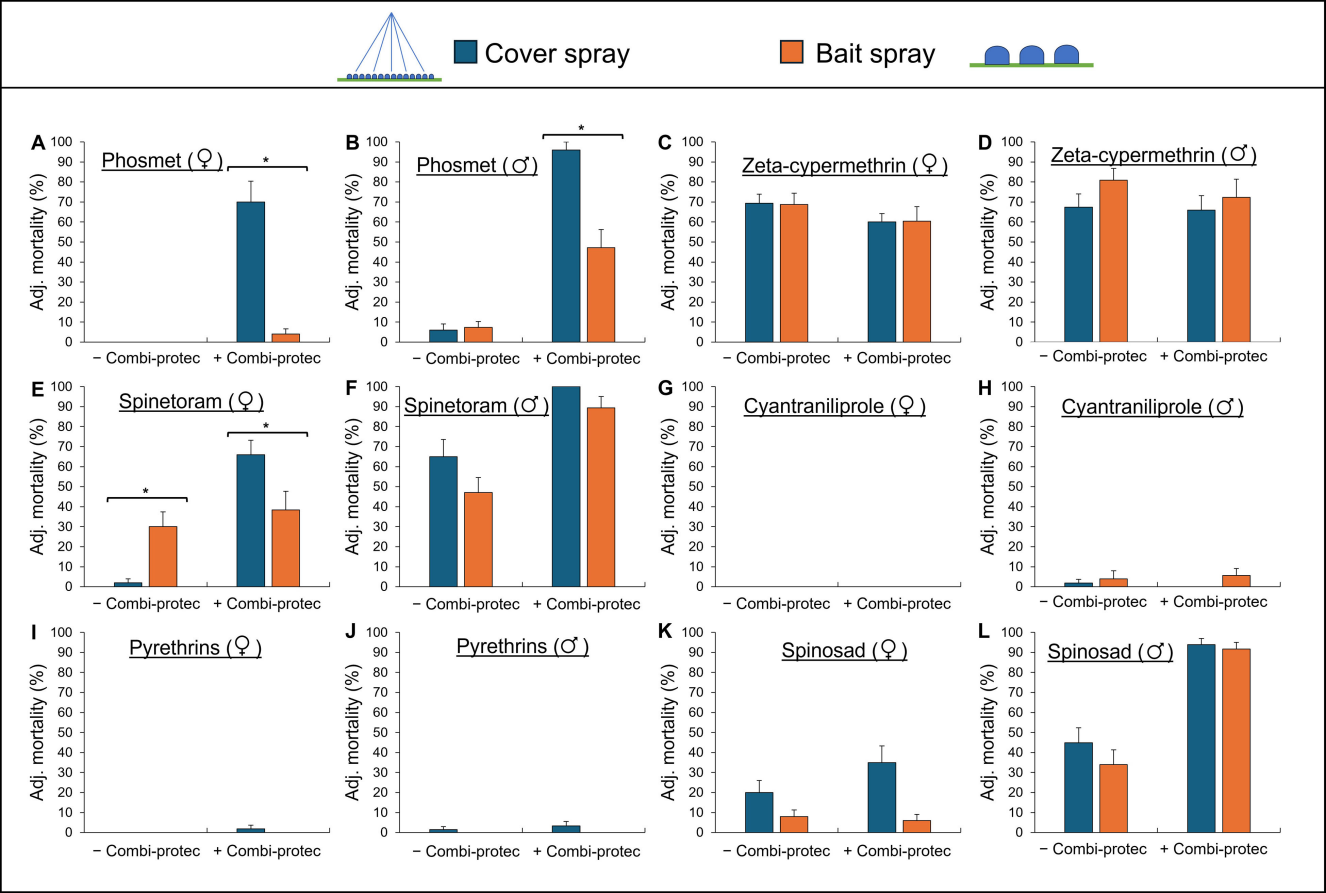
Effects of spray type and Combi-protec^®^ on female **(A, C, E, G, I, K)** and male **(B, D, F, H, J, L)** *Ganaspis kimorum* 24 h mortality at 3 days after treatment (DAT) for phosmet **(A, B)**, zeta-cypermethrin **(C, D)**, spinetoram **(E, F)**, cyantraniliprole **(G, H)**, pyrethrins **(I, J)**, and spinosad **(K, L)** sprayed at high rates (full, cover spray or 100%, bait spray). Asterisks (*) indicate a significant difference between treatments (*p* < 0.05).

**Figure 6:**
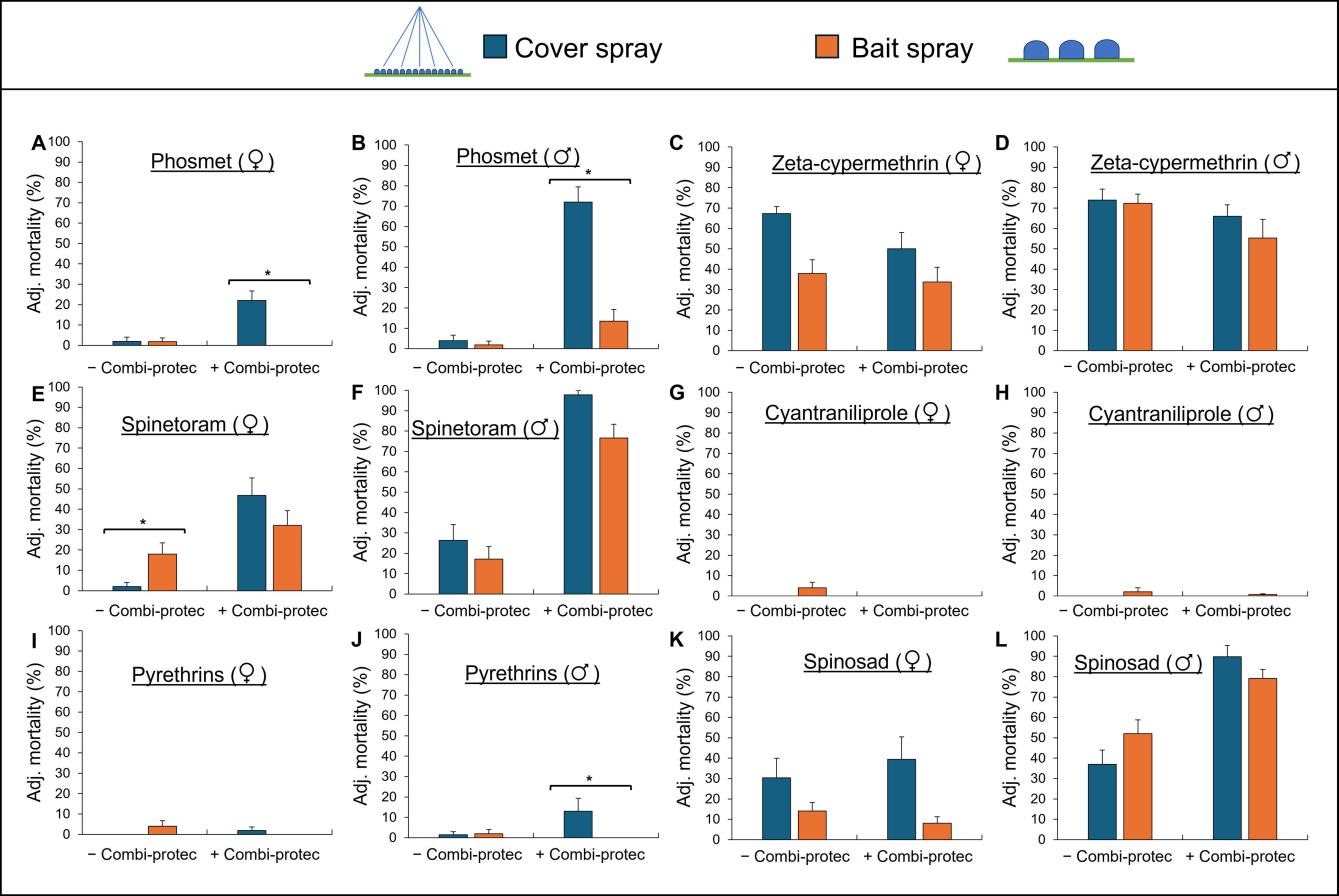
Effects of spray type and Combi-protec^®^ on female **(A, C, E, G, I, K)** and male **(B, D, F, H, J, L)** *Ganaspis kimorum* 24 h mortality at 3 days after treatment (DAT) for phosmet **(A, B)**, zeta-cypermethrin **(C, D)**, spinetoram **(E, F)**, cyantraniliprole **(G, H)**, pyrethrins **(I, J)**, and spinosad **(K, L)** sprayed at intermediate rates (half, cover spray or 50%, bait spray). Asterisks (*) indicate a significant difference between treatments (*p* < 0.05).

**Figure 7:**
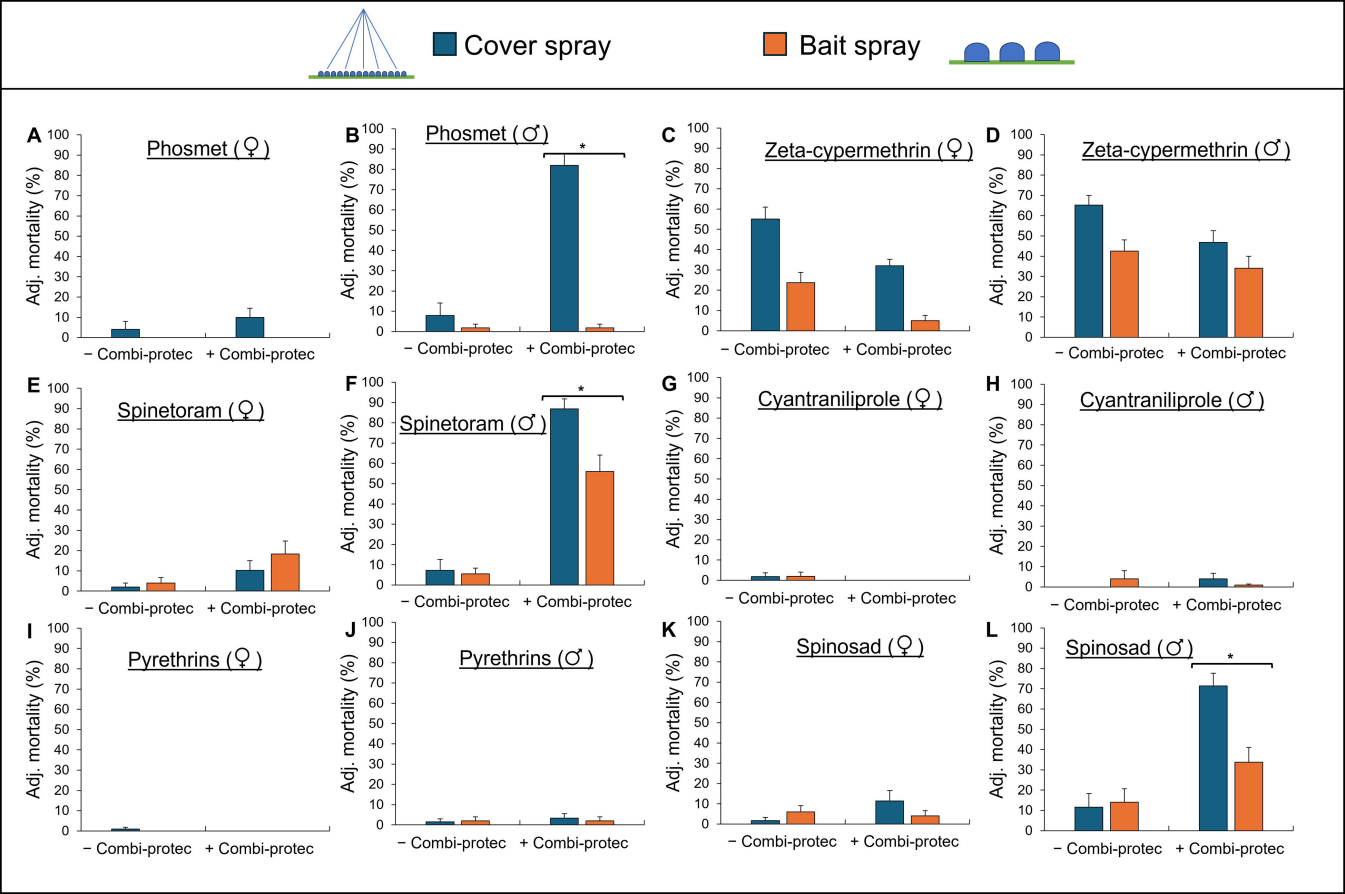
Effects of spray type and Combi-protec^®^ on female **(A, C, E, G, I, K)** and male **(B, D, F, H, J, L)** *Ganaspis kimorum* 24 h mortality at 3 days after treatment (DAT) for phosmet **(A, B)**, zeta-cypermethrin **(C, D)**, spinetoram **(E, F)**, cyantraniliprole **(G, H)**, pyrethrins **(I, J)**, and spinosad **(K, L)** were sprayed at low rates (quarter, cover spray or 10%, bait spray). Asterisks (*) indicate a significant difference between treatments (*p* < 0.05).

Comparative lethality of each insecticide between cover and bait sprays, with and without Combi-protec^®^, at high, intermediate, and low rates on male and female *G. kimorum* is presented in **Supplementary Figures 5**–**S7** (0 DAT), **Figures 5**–**7** (3 DAT), and **Supplementary Figures 8**–**S10** (7 DAT). Regardless of the presence of Combi-protec^®^, bait sprays generally reduced insecticide lethality to *G. kimorum* compared to cover sprays, particularly at low rates for both males and females (**Figure 1** vs. **Figure 2**). When Combi-protec^®^ was included, cover sprays resulted in significantly higher mortality than bait sprays in 26 of 108 instances (24%; 6 insecticides × 2 sexes × 3 rates × 3 DATs), although the effect varied depending on insecticide type, application rate, DAT, and parasitoid sex (**Table 6**; **Figures 5**–**7**, **Supplementary Figures 5**–**10**). In combination with Combi-protec^®^, bait sprays reduced the lethality of phosmet in 11 instances, spinetoram in nine, and spinosad in four instances at lower rates and later DATs, relative to cover sprays. Similarly, cover sprays of pyrethrins with Combi-protec^®^ caused higher male mortality in two instances at intermediate and low rates at three and seven DAT (**Figure 6**; **Supplementary Figure 10**). Cover sprays of zeta-cypermethrin with Combi-protec^®^ also resulted in significantly higher mortality in one instance (**Supplementary Figure 7**). In contrast, in two instances bait sprays with Combi-protec^®^ were more lethal than cover sprays: spinosad at the high rate at 0 DAT was more lethal to males when applied as a bait spray (**Supplementary Figure 5**), and cyantraniliprole at the intermediate rate at 7 DAT caused higher mortality as a bait spray than as a cover spray (**Supplementary Figure 9**).

**Table 6:** Effects of insecticide type, Combi-protec^®^, spray type, rate, and days after treatment (DAT) on mortality of *Ganaspis kimorum* after 24 h of exposure in the laboratory.

Variables	χ^2^		df	*P* ^a^	
	Male	Female		Male	Female
Insecticide	268.03	122.21	5	**<0.001**	**<0.001**
Combi-protec	2.96	1.82	1	0.085	0.177
Spray	4.10	49.36	1	**0.043**	**<0.001**
Rate	104.11	36.94	2	**<0.001**	**<0.001**
DAT	65.82	6.42	2	**<0.001**	**0.040**
Insecticide × Combi-protec	7.42	22.38	5	0.191	**<0.001**
Insecticide × Spray	67.44	57.81	5	**<0.001**	**<0.001**
Combi-protec × Spray	2.29	3.58	1	0.130	0.059
Insecticide × Rate	105.51	52.55	10	**<0.001**	**<0.001**
Combi-protec × Rate	24.56	2.01	2	**<0.001**	0.366
Spray × Rate	37.97	2.46	2	**<0.001**	0.293
Insecticide × DAT	62.98	9.23	10	**<0.001**	0.511
Combi-protec × DAT	49.90	7.06	2	**<0.001**	**0.029**
Spray × DAT	26.69	57.71	2	**<0.001**	**<0.001**
Rate × DAT	35.75	6.9	4	**<0.001**	0.141
Insecticide × Combi-protec × Spray	22.74	52.12	5	**<0.001**	**<0.001**
Insecticide × Combi-protec × Rate	76.86	20.80	10	**<0.001**	**0.023**
Insecticide × Spray × Rate	56.54	26.10	10	**<0.001**	**0.004**
Combi-protec × Spray × Rate	7.02	2.29	2	**0.030**	0.318
Insecticide × Combi-protec × DAT	67.06	27.17	10	**<0.001**	**0.002**
Insecticide × Spray × DAT	58.08	48.11	10	**<0.001**	**<0.001**
Combi-protec × Spray × DAT	33.38	11.27	2	**<0.001**	**0.004**
Insecticide × Rate × DAT	87.80	15.42	20	**<0.001**	0.752
Combi-protec × Rate × DAT	21.96	2.28	4	**<0.001**	0.685
Spray × Rate × DAT	28.21	3.48	4	**<0.001**	0.482
Insecticide × Combi-protec × Spray × Rate	36.02	11.04	10	**<0.001**	0.355
Insecticide × Combi-protec × Spray × DAT	60.37	48.25	10	**<0.001**	**<0.001**
Insecticide × Combi-protec × Rate × DAT	71.47	21.95	20	**<0.001**	0.343
Insecticide × Spray × Rate × DAT	56.95	15.64	20	**<0.001**	0.797
Combi-protec × Spray × Rate × DAT	9.97	25.31	4	**0.041**	**<0.001**
Insecticide × Combi-protec × Spray × Rate × DAT	53.70	34.19	20	**<0.001**	**0.025**

^a^Bold indicates significant P-value (*p* < 0.05).

In contrast to patterns observed in the presence of Combi-protec^®^—where bait sprays generally reduced lethality relative to cover sprays—differences between application methods were less consistent in its absence (**Figures 5**–**7**; **Supplementary Figures 5**–**10**). Without Combi-protec^®^, cover sprays were significantly more lethal than bait sprays in six out of 108 instances (6%), whereas bait sprays were more lethal in eight instances (7%) (**Figures 5**–**7**; **Supplementary Figures 5**–**10**). Opposite outcomes were sometimes observed for the same insecticide depending on the rate and DAT. For example, without Combi-protec^®^, bait sprays of spinetoram were more lethal to females at the high and intermediate rates at 3 DAT (**Figures 5**, **6**) and at the high rate at 7 DAT (**Supplementary Figure 8**). However, cover sprays of the same insecticide were more lethal to females at low rates at 0 DAT (**Supplementary Figure 7**). Similarly, spinetoram bait sprays were significantly more lethal to males than cover sprays at high and intermediate rates at 7 DAT (**Supplementary Figures 8**, **9**), whereas cover sprays were more lethal at intermediate rates at 0 DAT (**Supplementary Figure 6**).

### 3.4 Do laboratory results translate to field conditions?

**Figure 8:**
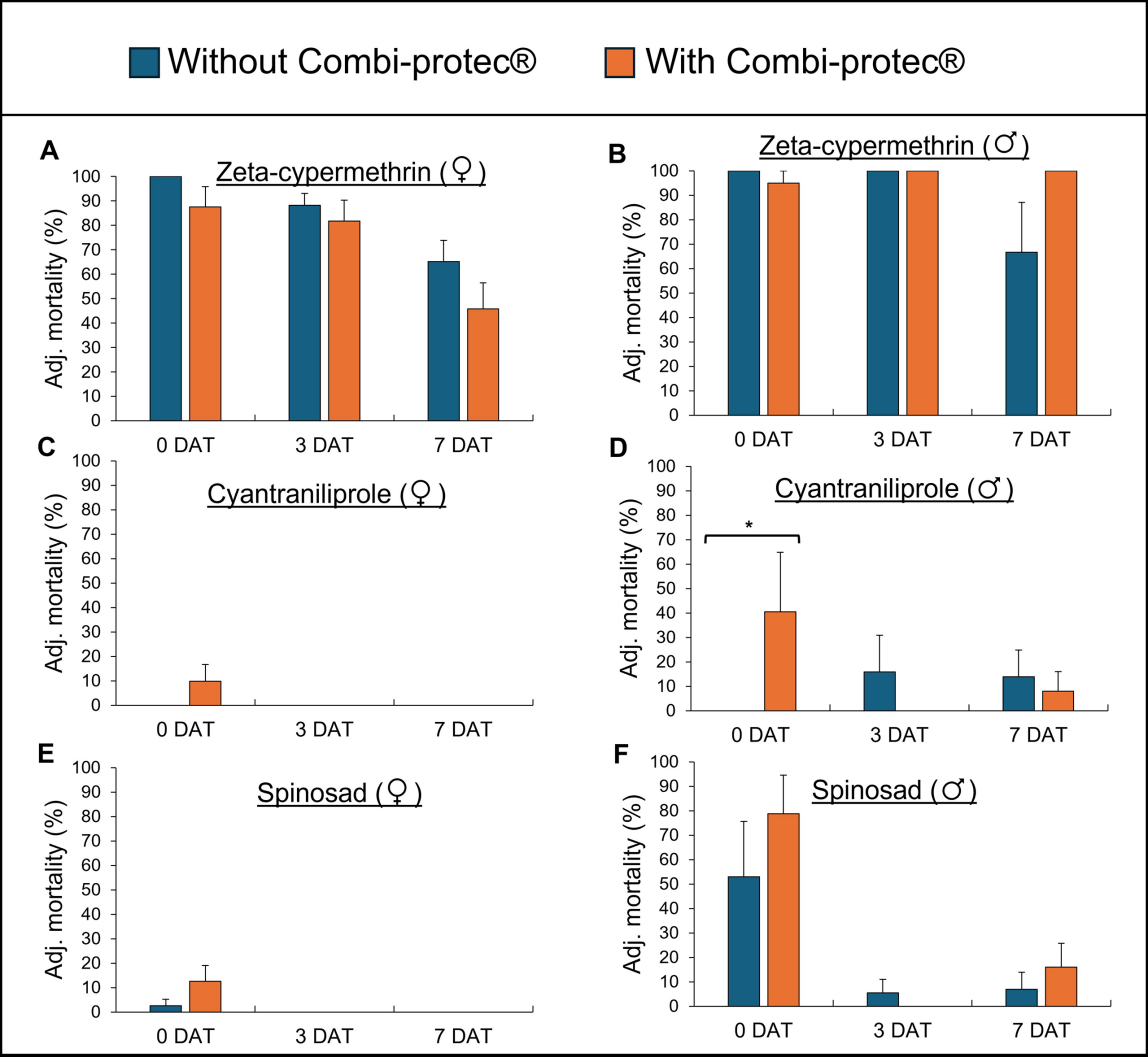
Effects of Combi-protec^®^ on female **(A, C, E)** and male **(B, D, F)** *Ganaspis kimorum* 72 h mortality when exposed to different days after treatment (DAT) of cover sprays of zeta-cypermethrin **(A, B)**, cyantraniliprole **(C, D)**, and spinosad **(E, F)** in the field. Letters indicate a significant difference between treatments (*p* < 0.05). Asterisks (*) indicate a significant difference between treatments (p < 0.05).

Generally, field-applied insecticides had effects on *G. kimorum* similar to those observed with laboratory-treated material, with zeta-cypermethrin consistently producing the highest mortality, followed by spinosad and then cyantraniliprole. As in laboratory assays, earlier DATs were generally more lethal than later ones. Notably, after 24 h of exposure to zeta-cypermethrin aged for 7 DAT under field conditions, the addition of Combi-protec^®^ significantly increased male mortality by 33% compared with the insecticide applied alone (**Supplementary Figure 11**). After 72 h of exposure, only cyantraniliprole at 0 DAT exhibited a significant interaction with Combi-protec^®^, increasing male mortality from 0% to approximately 40% (**Table 7**; **Figure 8**).

**Table 7:** Effects of insecticide type, Combi-protec^®^, and days after treatment (DAT) on mortality of *Ganaspis kimorum* after 72 h of exposure to a full rate and cover spray conditions in the field.

Variables	χ^2^		df	*P* ^a^	
	Male	Female		Male	Female
Insecticide	35.17	283.42	2	**<0.001**	**<0.001**
Combi-protec	1.81	3.68	1	0.178	0.055
DAT	13.74	0.38	2	**0.001**	0.825
Insecticide × Combi-protec	4.97	7.14	2	0.083	**0.028**
Insecticide × DAT	14.28	9.96	4	**0.006**	**0.041**
Combi-protec × DAT	1.69	2.45	2	0.431	0.293
Insecticide × Combi-protec × DAT	7.86	2.34	4	0.097	0.674

^a^Bold indicates significant P-value (*p* < 0.05).

## 4 Discussion

*Drosophila suzukii* management has relied on conventional insecticides since its emergence as a major globally invasive species (Asplen et al., 2015; Tait et al., 2021). Insecticides such as spinosyns (spinosad for organic and spinetoram for conventional systems), anthranilic diamides (cyantraniliprole), pyrethroids (zeta-cypermethrin), pyrethrins (organic systems), and organophosphates (phosmet) are often used to control *D. suzukii*. However, their repeated and intensive use can lead to ecological consequences, including the development of resistance, secondary pest outbreaks, and harm to non-target species (e.g., natural enemies and pollinators) (Fitt, 2000; Sparks et al., 2019). These outcomes could undermine the benefits of insecticide applications, leading to increased crop damage and economic losses. Integrating alternative strategies, such as behavioral and biological control, can reduce reliance on chemical treatments, although these approaches may occasionally interact antagonistically. Here, we integrate chemical, behavioral, and biological control strategies for *D. suzukii*.

This study demonstrated that insecticides used against *D. suzukii* differentially affect male and female adult *G. kimorum*, resulting in varying levels of mortality, with a general ranking from highest to lowest: zeta-cypermethrin > phosmet = spinetoram = spinosad > cyantraniliprole = pyrethrins. In general, males were more sensitive than females to these insecticides, particularly to phosmet. Since there are no clear differences in body size (Stahl et al., 2024), other factors may explain this disparity, such as higher mobility or activity levels among males (Andreazza et al., 2020). Among the six insecticides evaluated, zeta-cypermethrin caused the highest mortality and should be considered high-risk for this beneficial parasitoid. This result was expected, as pyrethroids act on neural targets conserved across a broad range of insects, and their non-target effects are well documented (Bayram et al., 2010; Ahmad et al., 2012; Palmquist et al., 2012; Lisi et al., 2024a). Phosmet, spinetoram, and spinosad also negatively affected *G. kimorum*, but their impact depended on application rate and residual activity. In contrast, cyantraniliprole and pyrethrins caused the lowest mortality and can be considered lower-risk options, consistent with previous research (Roubos et al., 2019; Lisi et al., 2024a). Cyantraniliprole exhibited reduced lethality to *G. kimorum*, likely due to its selective mode of action targeting ryanodine receptors in muscle tissue (Sparks and Nauen, 2015; Peng et al., 2025). Interestingly, cyantraniliprole is highly lethal to *D. suzukii* when applied alone (Shaw et al., 2019) and when incorporated into baits (Noble et al., 2021), but it exhibits low toxicity to its parasitoid, *G. kimorum* (Fellin et al., 2024; Lisi et al., 2024a). A similar pattern has been reported for the Asian citrus psyllid, *Diaphorina citri* Kuwayama (Hemiptera: Psyllidae), and its parasitoid, *Tamarixia radiata* (Waterston) (Hymenoptera: Eulophidae) (Tiwari and Stelinski, 2012). This selectivity makes cyantraniliprole a promising candidate for integration into IPM programs aimed at conserving *G. kimorum* in conventional production systems.

Incorporating the phagostimulant Combi-protec^®^ into insecticide formulations enhances ingestion by adult *D. suzukii*, thereby improving efficacy while reducing the amount of active ingredient required for effective control (Babu et al., 2022; Ferguson et al., 2025). Lower application rates may mitigate ecological risks associated with conventional non-baited application rates (Knight et al., 2016; Babu et al., 2022; Escobedo-Quevedo et al., 2024). However, just as insecticides can harm non-target species, Combi-protec^®^ can also stimulate feeding by parasitoids such as *G. kimorum*, potentially increasing their exposure and mortality. The addition of Combi-protec^®^ significantly increased *G. kimorum* mortality when combined with spinosad and spinetoram, two insecticides primarily absorbed through ingestion. Its inclusion with phosmet, which acts via both contact and ingestion, also elevated mortality. In contrast, combining Combi-protec^®^ with zeta-cypermethrin, a primarily contact insecticide, resulted in a less pronounced increase in mortality, underscoring the importance of absorption route in determining non-target effects. Piovesan et al. (2023) also reported differential lethal effects of bait–insecticide combinations on the fruit fly parasitoid *Doryctobracon areolatus* (Szépligeti) (Hymenoptera: Braconidae), with Malathion^®^ 1000 EC-based baits being more harmful than those containing phosmet. Our findings support the conclusion that Combi-protec^®^ stimulates feeding behavior not only in *D. suzukii* (Babu et al., 2022), but also in *G. kimorum*. Thus, although Combi-protec^®^ enhances insecticide performance against *D. suzukii*, its compatibility with biological control agents must be carefully considered prior to use.

Lethality of phosmet, spinosad, and spinetoram to *G. kimorum* tended to be lower when these insecticides were applied as bait sprays rather than as cover sprays. Bait formulations limited the treated area within the arena, potentially reducing insecticide contact and thus the lethal effects on the wasps. This reduced exposure likely explains the lower overall toxicity of bait sprays to *G. kimorum* and the smaller increase in mortality observed when Combi-protec^®^ was included. Laboratory and field studies by Fountain et al. (2025) demonstrated that bait applications combined with insecticides targeting *D. suzukii* were no more toxic to non-target insects than standard full-foliar applications. There were instances where Combi-protec^®^ reduced the lethality of certain insecticides to *G. kimorum*, such as zeta-cypermethrin. While the exact mechanism is unknown, we speculate that feeding on the sugar-rich phagostimulant may either reduce overall insecticide ingestion or enhance energy metabolism, thereby increasing the activity of detoxification enzymes. In this study, we evaluated only lethal effects on *G. kimorum*; future research should examine whether wasps exposed to insecticides, with and without Combi-protec^®^, exhibit reduced parasitism. For example, Lisi et al. (2024b) reported sublethal effects of cyantraniliprole on the survival and fertility of *Trichopria drosophilae* Perkins (Hymenoptera: Diapriidae), a pupal parasitoid of *D. suzukii*.

Compared to laboratory conditions, insecticides in the field are exposed to environmental factors such as sunlight (UV radiation), temperature fluctuations, rainfall, and wind, all of which can reduce their persistence and effectiveness. Our semi-field experiment controlled most of these factors, except for UV exposure, which likely explains the differences observed relative to the laboratory setting. In both bioassays, zeta-cypermethrin was highly lethal to *G. kimorum*, whereas cyantraniliprole exhibited low lethality, and the addition of Combi-protec^®^ had minimal effect. Similarly, Leach and Wilson (2023) reported higher lethality of zeta-cypermethrin than cyantraniliprole on *G. kimorum* in semi-field studies conducted on tart cherries. Notably, spinosad was more lethal in the laboratory than in the field, particularly to females, and its activity was not influenced by the addition of Combi-protec^®^.

Understanding how Combi-protec^®^ affects *G. kimorum* mortality enables more informed decisions on insecticide selection and application methods. The phagostimulant may enhance efficacy the most against *D. suzukii* when used with insecticides that are primarily absorbed through ingestion, but doing so also poses a greater risk to parasitoids. When parasitoid activity is high, combining Combi-protec^®^ with reduced-risk contact-acting insecticides may help minimize non-target impacts. Cyantraniliprole and pyrethrins emerge as promising lower-risk options for conventional and organic systems, respectively. Despite cyantraniliprole’s ingestion-based uptake, it caused low mortality in both laboratory and semi-field bioassays, likely due to its selective mode of action. The use of insecticides with selective modes of action can therefore help to conserve released *G. kimorum* populations. In our laboratory arenas, the benefits of the bait formulation were observed primarily when the phagostimulant was included. We expect that, under field conditions, applying small amounts of insecticide to limited areas for *D. suzukii* may be ineffective without a phagostimulant. Due to logistical constraints—such as achieving consistent coverage with small droplets on individual bushes—we employed only cover sprays under semi-field conditions. Future studies are needed to confirm these effects under commercial field conditions.

## 5 Conclusions

Among the insecticides tested, cyantraniliprole and pyrethrins resulted in the lowest *G. kimorum* mortality. Although Combi-protec^®^ increased mortality when combined with phosmet, spinosad, and spinetoram, these negative effects were mitigated when formulations were applied as bait sprays rather than cover sprays. In contrast, zeta-cypermethrin remained highly toxic to *G. kimorum* regardless of the addition of Combi-protec^®^ or application method. Ultimately, selecting insecticides based on mode of action, particularly when used alongside behavioral modifiers such as Combi-protec^®^, can help refine pest management strategies. These insights are critical for reducing antagonism among chemical, behavioral, and biological controls and for promoting a more sustainable approach to managing *D. suzukii* in small fruit crops.

## Data Availability

The raw data supporting the conclusions of this article will be made available by the authors, without undue reservation.
