# Correction: Compatibility of insecticides and a phagostimulant with *Ganaspis kimorum*, a parasitoid of *Drosophila suzukii*

**DOI:** 10.3389/fpls.2026.1928541

**Published:** 2026-07-30

**Authors:** Jack Collins, Yahel Ben-Zvi, John McCulloch, Ashfaq A. Sial, Robert Holdcraft, Philip Fanning, Rufus Isaacs, Elena Rhodes, Oscar Liburd, Cesar Rodriguez-Saona

**Affiliations:** 1 Philip E. Marucci Center for Blueberry & Cranberry Research and Extension (PE) Marucci Center, Rutgers University, Chatsworth, NJ, United States; 2 Department of Entomology, University of Georgia, Athens, GA, United States; 3 School of Biology and Ecology, University of Maine, Orono, ME, United States; 4 Department of Entomology, Michigan State University, East Lansing, MI, United States; 5 Entomology and Nematology Department, University of Florida, Gainesville, FL, United States

**Keywords:** behavioral manipulation, biological control, Combi-protec^®^, integrated pest management, invasive pest, spotted-wing drosophila

In the **Acknowledgements** statement, we omitted to thank Arun Babu and Cera Jones.

“We thank the team at the Phillip Alampi Beneficial Insect Laboratory, particularly Angela Lovero, Jon Beetle, and Alexandra Gillett, for supplying the wasps. We thank Arden Lambert, Stella Ruber, and Alexis Zapata for maintaining the wasp colony. We thank Arun Babu and Cera Jones for their help with data collection, and Betsy Beers, Frank Zalom, and Mary Rogers for their contributions to early discussions on experimental design. We also thank Chloe Hawkings and Changlu Wang for their helpful comments on an earlier draft of the manuscript”.

The original version of this article has been updated.

