# Supplementary material for: Compatibility of insecticides and a phagostimulant with Ganaspis kimorum, a parasitoid of Drosophila suzukii
Source: Front Plant Sci. 2026 May 29;17:1840796. doi: 10.3389/fpls.2026.1840796 (PMC13259691; doi:10.3389/fpls.2026.1840796)

Supplementary Figure S1. Effects of cover sprays at full (A, B), half (C, D), and quarter (E, F) rates and Combi-protec^®^ on female (A, C, E) and male (B, D, F) *Ganaspis kimorum* 24-h mortality at 0 days after treatment (DAT). Asterisks (*) indicate a significant difference between treatments (*p* < 0.05).


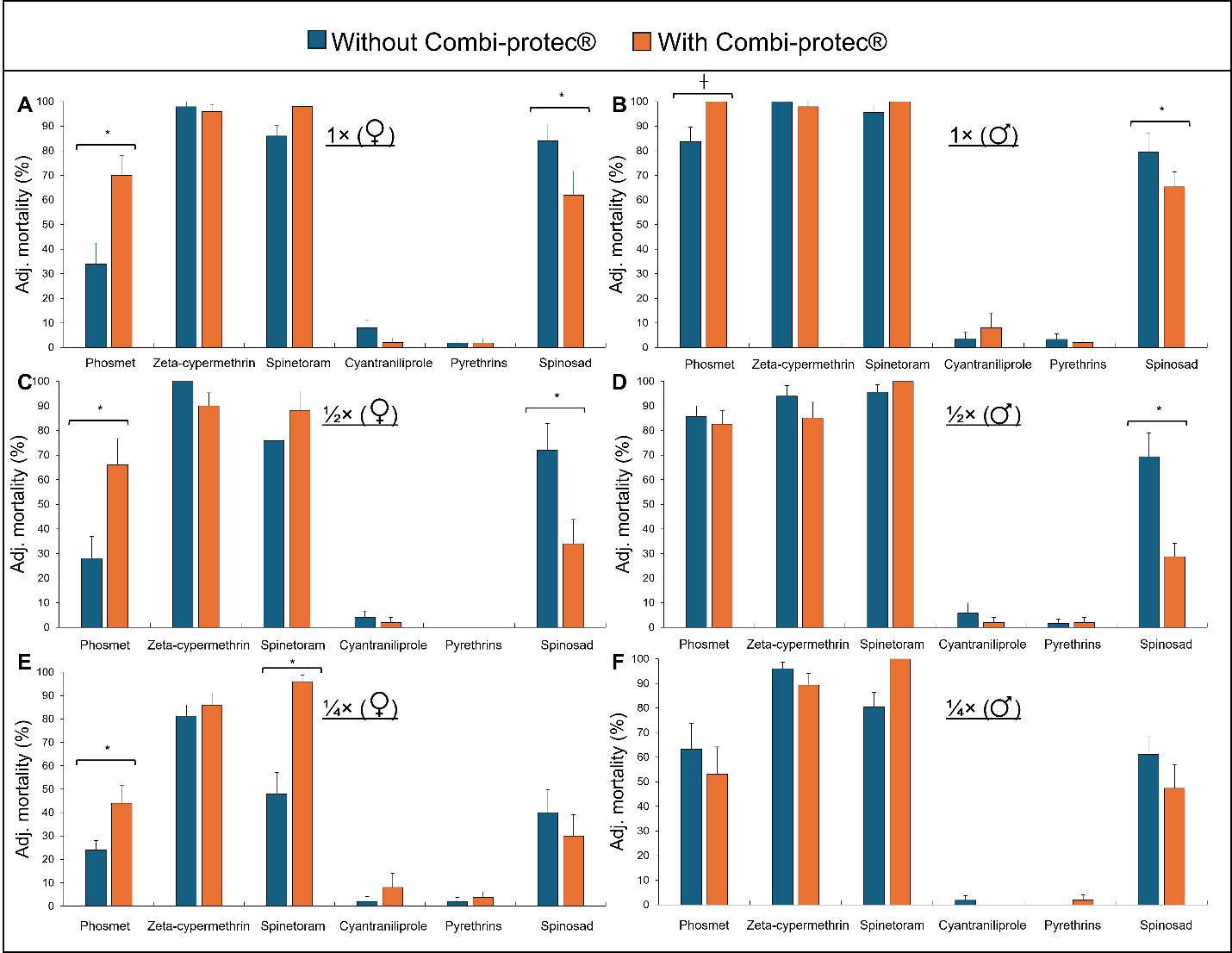


Supplementary Figure S2. Effects of bait sprays at 100% (A, B), 50% (C, D), and 10% (E, F) and Combi-protec^®^ on female (A, C, E) and male (B, D, F) *Ganaspis kimorum* 24-h mortality at 0 days after treatment (DAT). Asterisks (*) indicate a significant difference between treatments (*p* < 0.05).


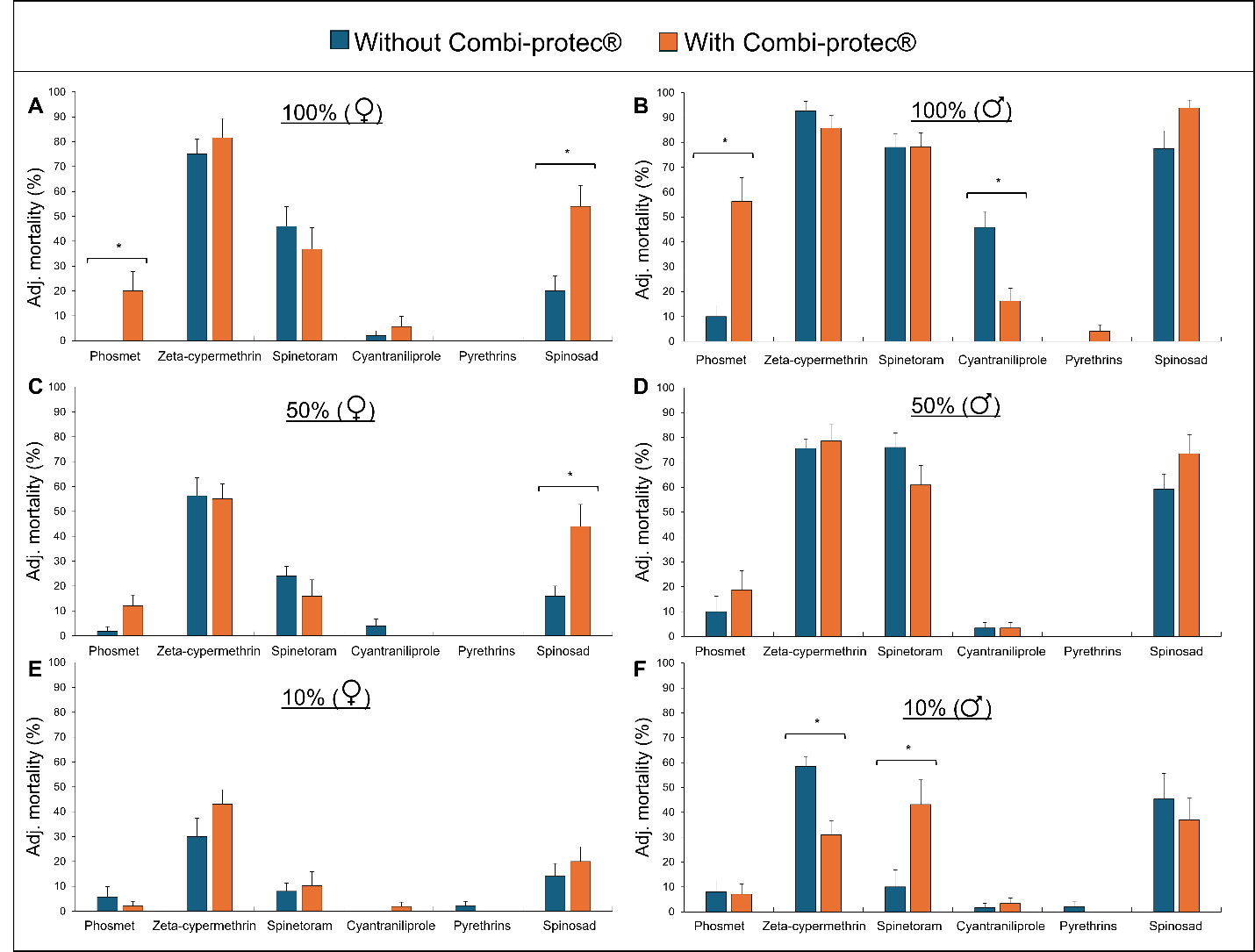


Supplementary Figure S3. Effects of cover sprays at full (A, B), half (C, D), and quarter (E, F) rates and Combi-protec^®^ on female (A, C, E) and male (B, D, F) *Ganaspis kimorum* 24-h mortality at 7 days after treatment (DAT). Asterisks (*) indicate a significant difference between treatments (*p* < 0.05).


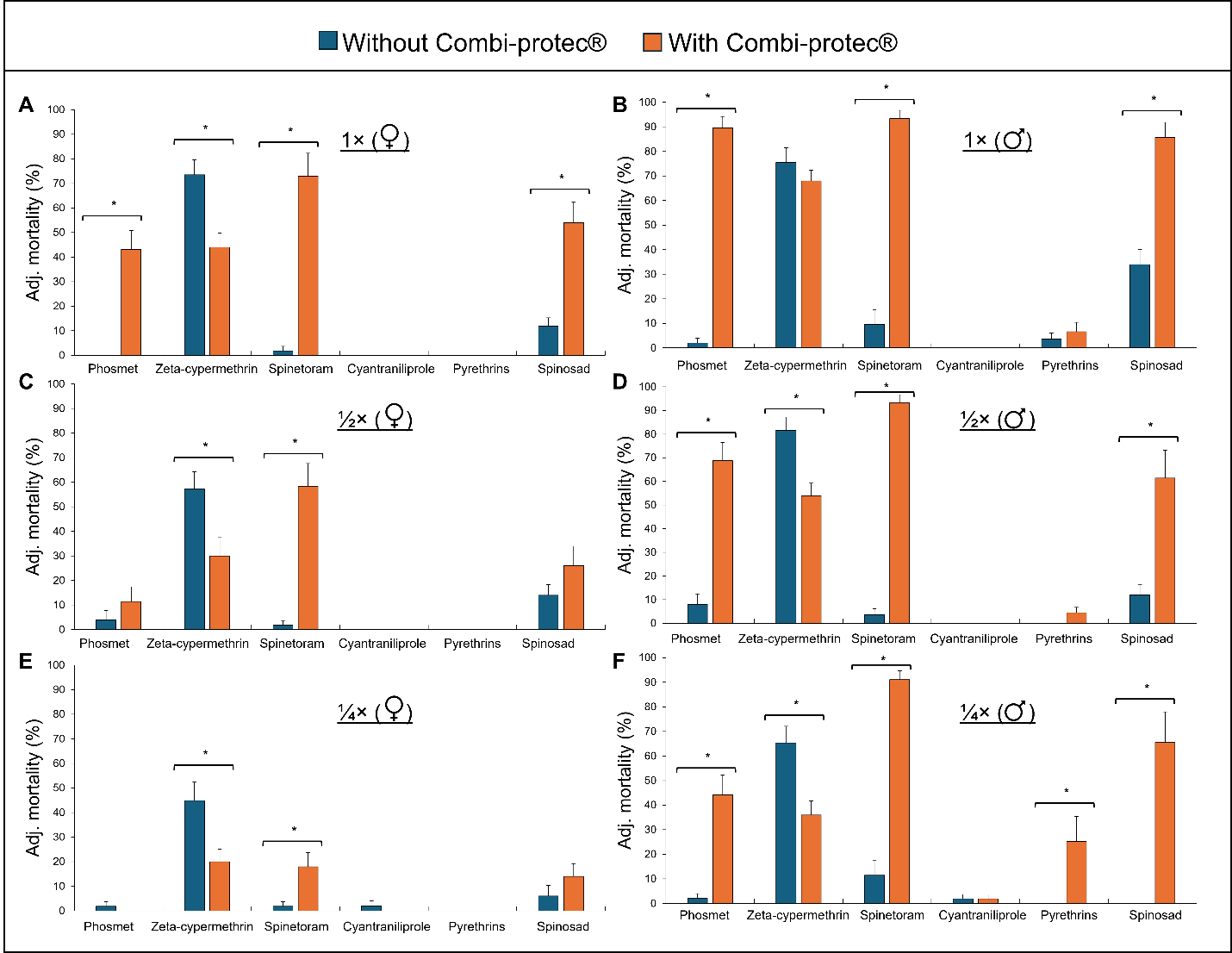


Supplementary Figure S4. Effects of bait sprays at 100% (A, B), 50% (C, D), and 10% (E, F) and Combi-protec^®^ on female (A, C, E) and male (B, D, F) *Ganaspis kimorum* 24-h mortality at 7 days after treatment (DAT). Asterisks (*) indicate a significant difference between treatments (*p* < 0.05).


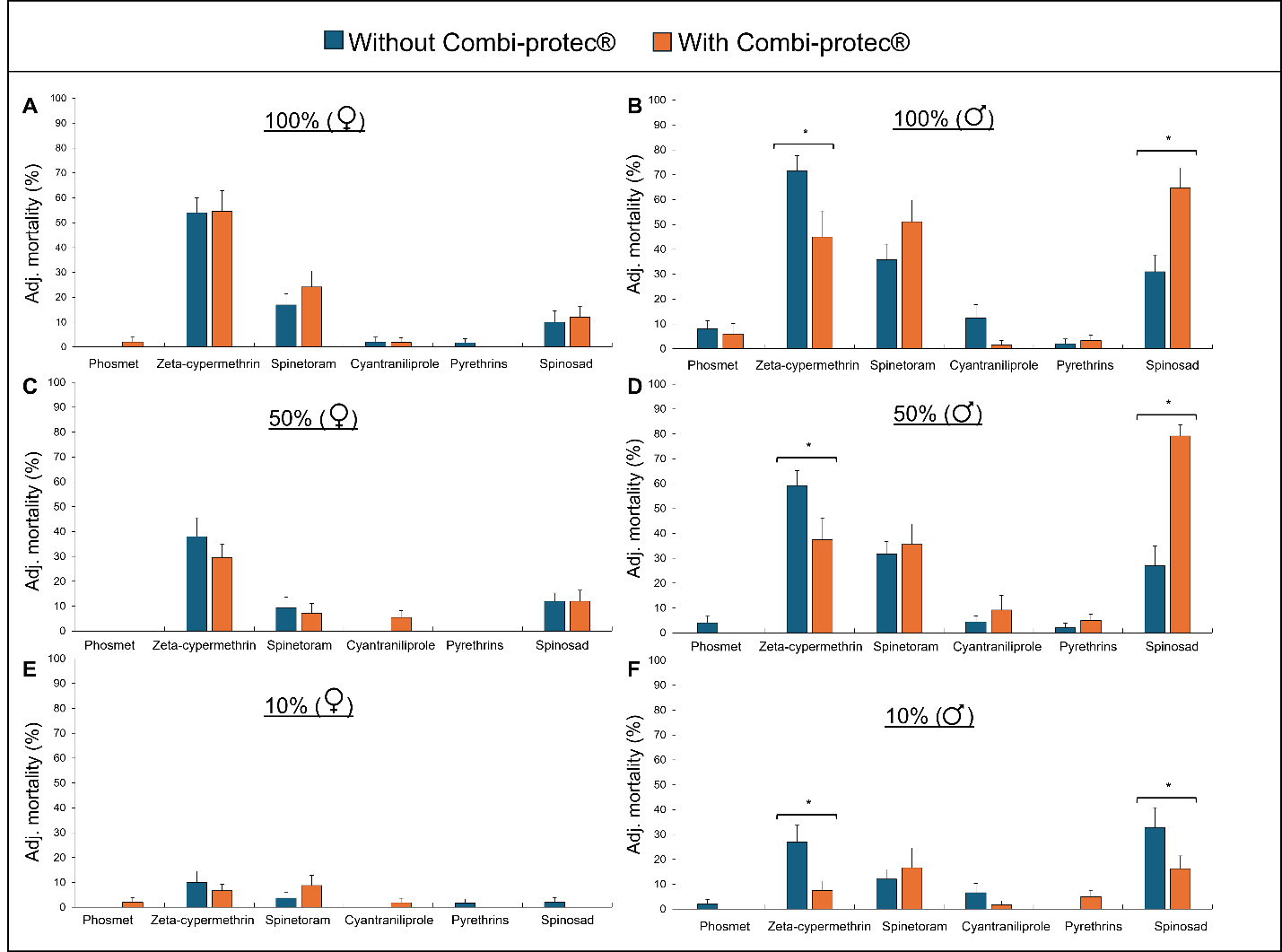


Supplementary Figure S5. Effects of spray type and Combi-protec^®^ on female (A, C, E, G, I, K) and male (B, D, F, H, J, L) *Ganaspis kimorum* 24-h mortality at 0 days after treatment (DAT) for phosmet (A, B), zeta-cypermethrin (C, D), spinetoram (E, F), cyantraniliprole (G, H), pyrethrins (I, J), and spinosad (K, L) sprayed at high rates (full, cover spray or 100%, bait spray). Asterisks (*) indicate a significant difference between treatments (*p* < 0.05).


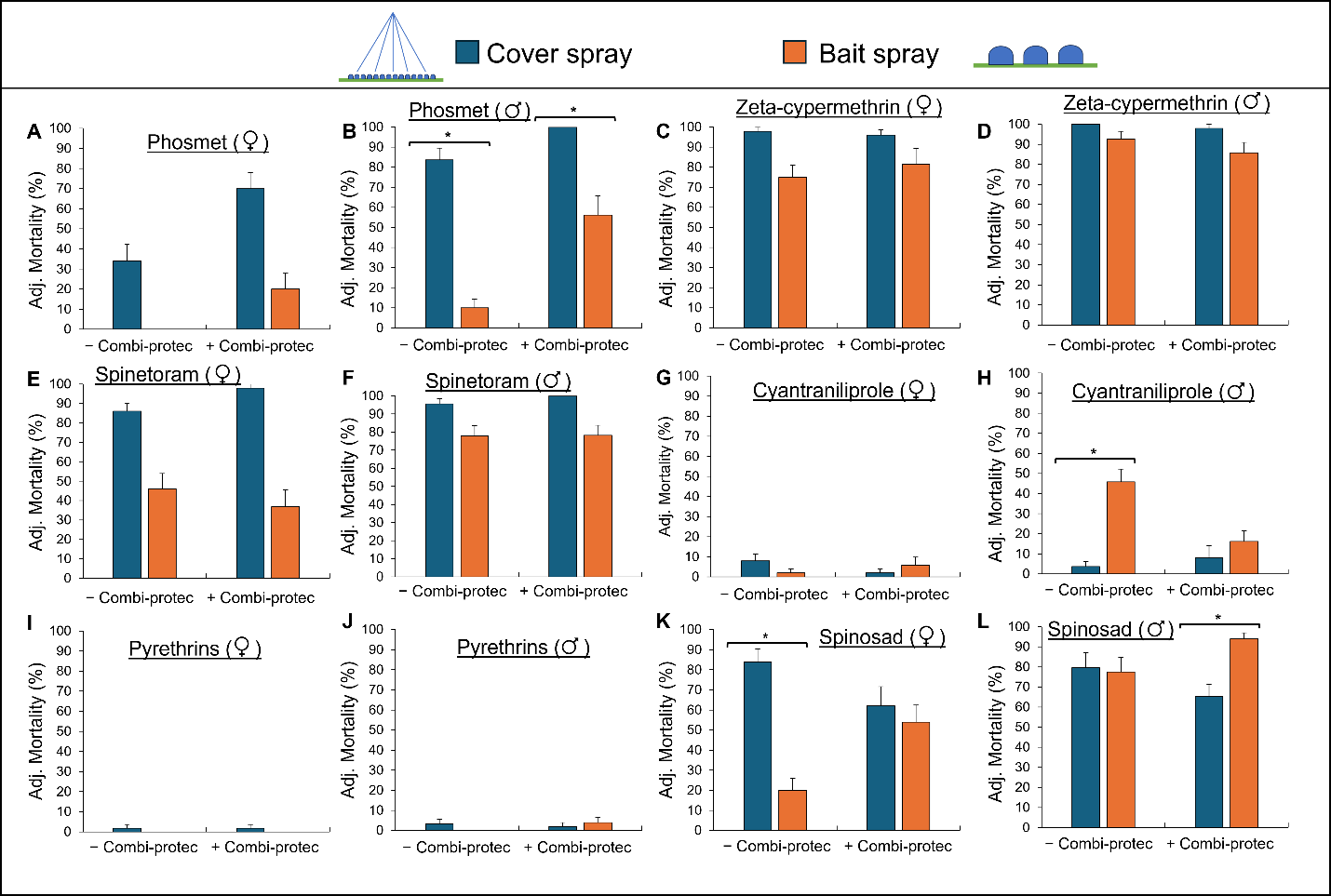


Supplementary Figure S6. Effects of spray type and Combi-protec^®^ on female (A, C, E, G, I, K) and male (B, D, F, H, J, L) *Ganaspis kimorum* 24-h mortality at 0 days after treatment (DAT) for phosmet (A, B), zeta-cypermethrin (C, D), spinetoram (E, F), cyantraniliprole (G, H), pyrethrins (I, J), and spinosad (K, L) sprayed at intermediate rates (half, cover spray or 50%, bait spray). Asterisks (*) indicate a significant difference between treatments (*p* < 0.05), crosses (┼) indicate a marginal differences between treatments (0.05 < *p* < 0.07).


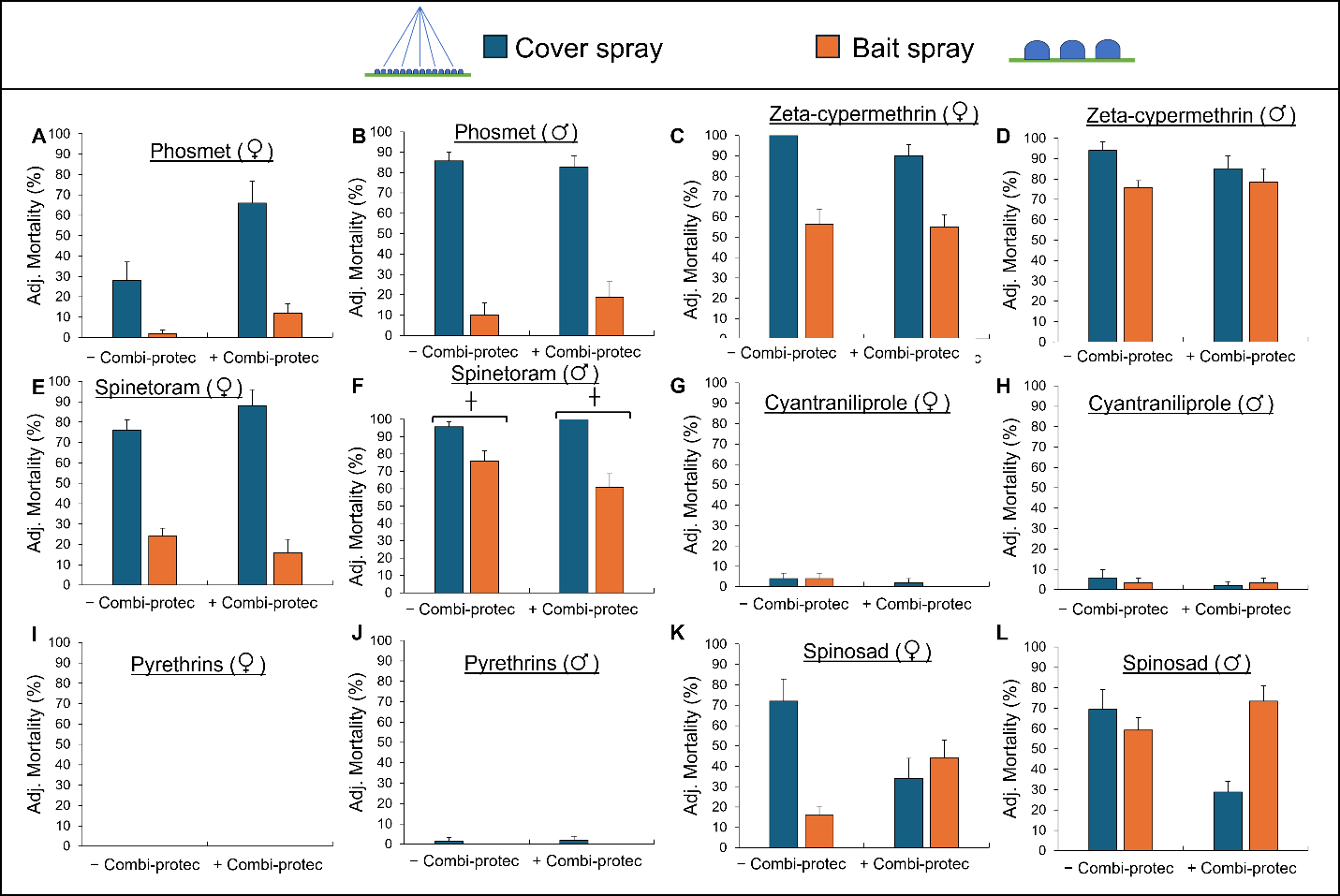


Supplementary Figure S7. Effects of spray type and Combi-protec^®^ on female (A, C, E, G, I, K) and male (B, D, F, H, J, L) *Ganaspis kimorum* 24-h mortality at 0 days after treatment (DAT) for phosmet (A, B), zeta-cypermethrin (C, D), spinetoram (E, F), cyantraniliprole (G, H), pyrethrins (I, J), and spinosad (K, L) were sprayed at low rates (quarter, cover spray or 10%, bait spray). Asterisks (*) indicate a significant difference between treatments (*p* < 0.05).


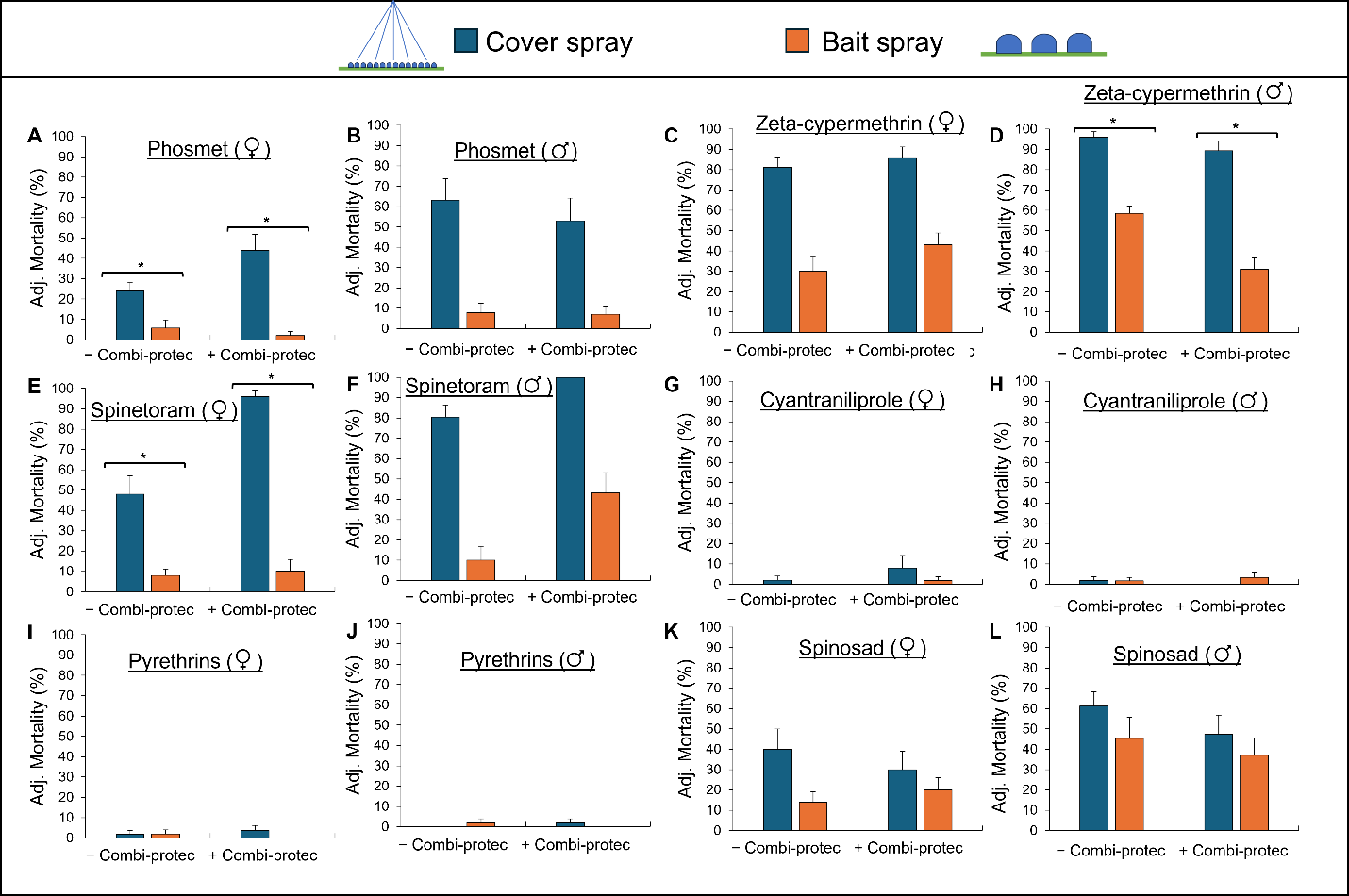


Supplementary Figure S8. Effects of spray type and Combi-protec^®^ on female (A, C, E, G, I, K) and male (B, D, F, H, J, L) *Ganaspis kimorum* 24-h mortality at 7 days after treatment (DAT) for phosmet (A, B), zeta-cypermethrin (C, D), spinetoram (E, F), cyantraniliprole (G, H), pyrethrins (I, J), and spinosad (K, L) sprayed at high rates (full, cover spray or 100%, bait spray). Asterisks (*) indicate a significant difference between treatments (*p* < 0.05).


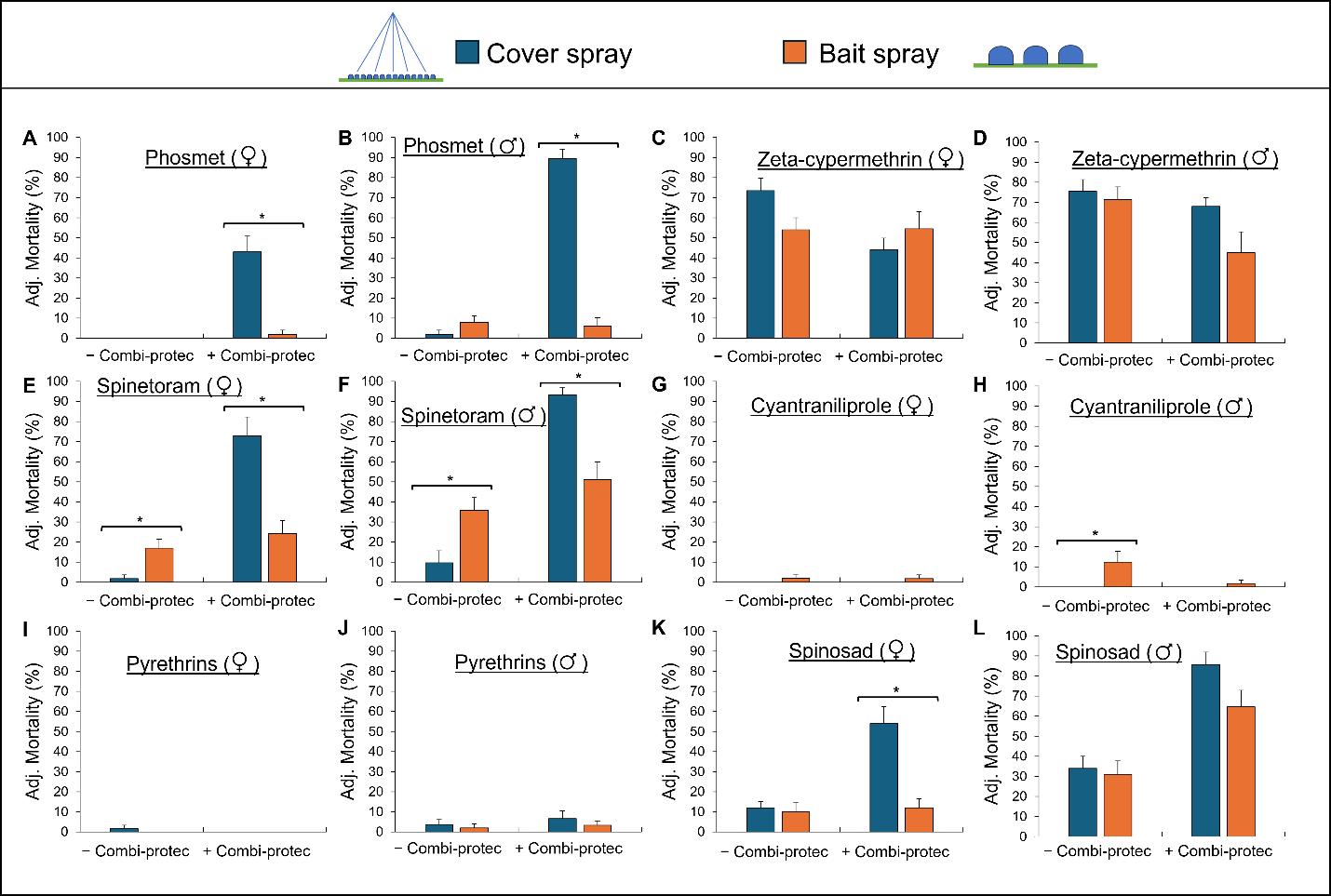


Supplementary Figure S9. Effects of spray type and Combi-protec^®^ on female (A, C, E, G, I, K) and male (B, D, F, H, J, L) *Ganaspis kimorum* 24-h mortality at 7 days after treatment (DAT) for phosmet (A, B), zeta-cypermethrin (C, D), spinetoram (E, F), cyantraniliprole (G, H), pyrethrins (I, J), and spinosad (K, L) sprayed at intermediate rates (half, cover spray or 50%, bait spray). Asterisks (*) indicate a significant difference between treatments (*p* < 0.05).


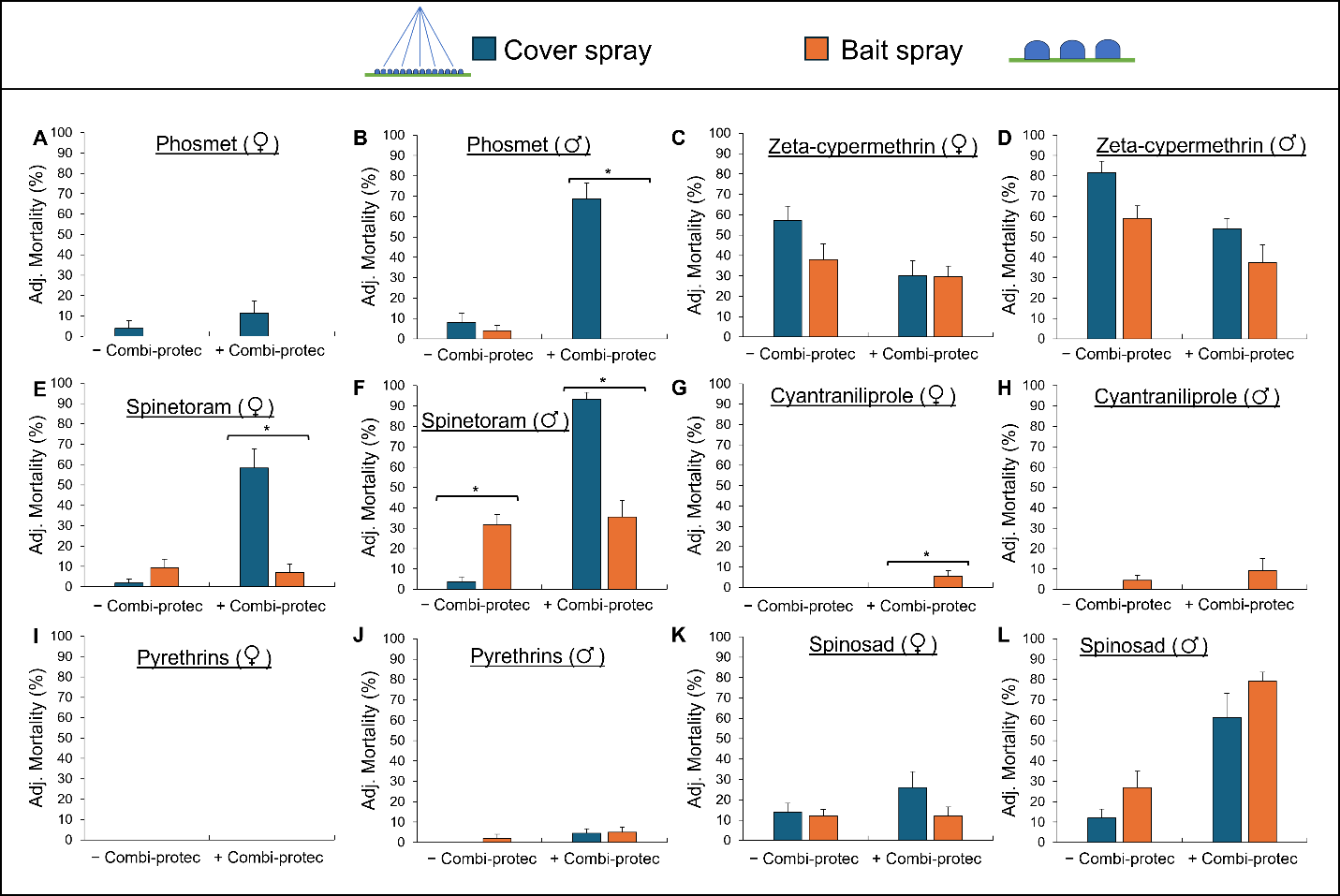


Supplementary Figure S10. Effects of spray type and Combi-protec^®^ on female (A, C, E, G, I, K) and male (B, D, F, H, J, L) *Ganaspis kimorum* 24-h mortality at 7 days after treatment (DAT) for phosmet (A, B), zeta-cypermethrin (C, D), spinetoram (E, F), cyantraniliprole (G, H), pyrethrins (I, J), and spinosad (K, L) were sprayed at low rates (quarter, cover spray or 10%, bait spray). Asterisks (*) indicate a significant difference between treatments (*p* < 0.05), crosses (┼) indicate a marginal differences between treatments (0.05 < *p* < 0.07).


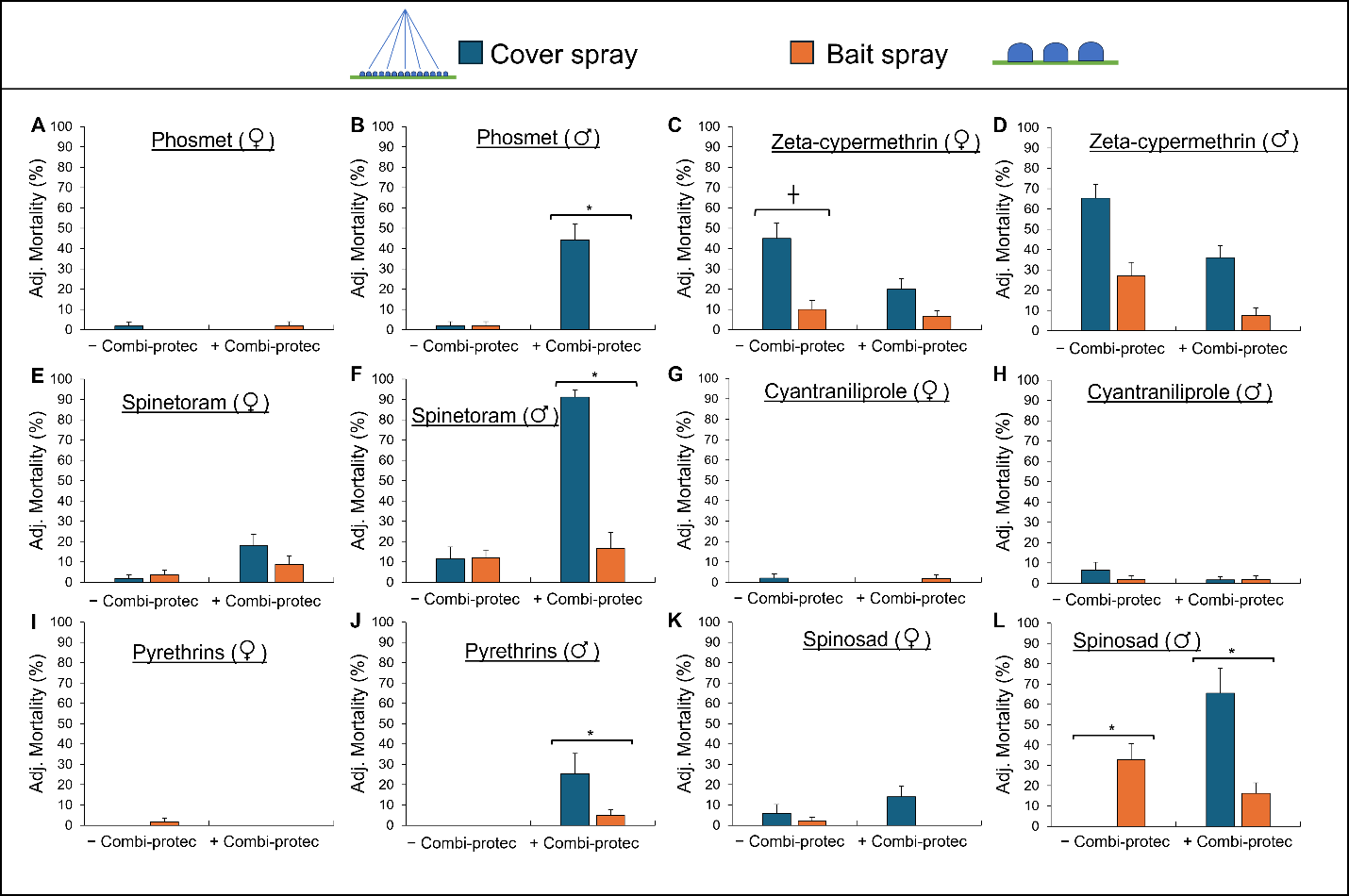


Supplementary Figure S11. Effects of Combi-protec^®^ on female (A, C, E) and male (B, D, F) *Ganaspis kimorum* 24-h mortality when exposed to different days after treatment (DAT) of cover sprays of zeta-cypermethrin (A, B), cyantraniliprole (C, D), and spinosad (E, F) in the field. Letters indicate a significant difference between treatments (*p* < 0.05).


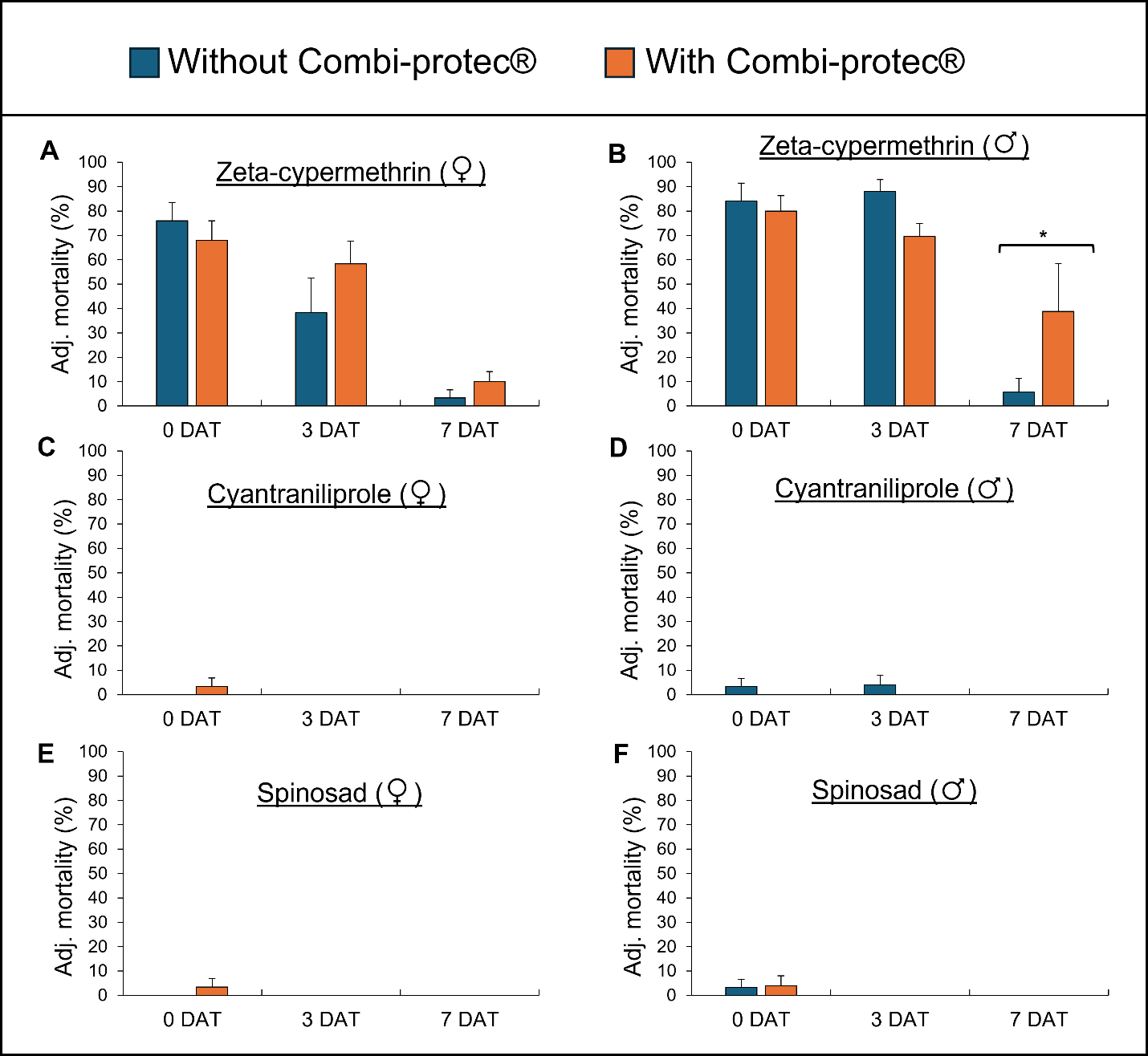

Supplement: Supplementary file 1 [file Table1.docx]
